# POMBOX: A Fission Yeast Cloning Toolkit for Molecular and Synthetic Biology

**DOI:** 10.1021/acssynbio.3c00529

**Published:** 2023-11-22

**Authors:** Téo Hebra, Helena Smrčková, Büsra Elkatmis, Martin Převorovský, Tomáš Pluskal

**Affiliations:** † Institute of Organic Chemistry and Biochemistry of the Czech Academy of Sciences, 160 00 Prague, Czech Republic; ‡ Department of Cell Biology, Faculty of Science, Charles University, 128 00 Prague, Czech Republic

**Keywords:** Schizosaccharomyces pombe, Golden Gate, MoClo, toolkit, plasmid, genomic integration

## Abstract

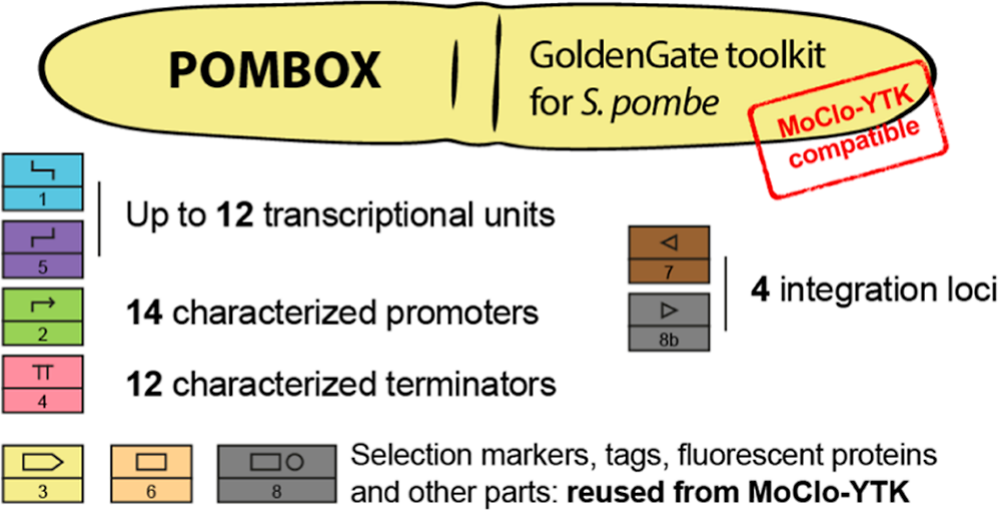

The fission yeast *Schizosaccharomyces pombe* is a popular model organism in molecular biology and cell physiology. With its ease of genetic manipulation and growth, supported by in-depth functional annotations in the PomBase database and genome-wide metabolic models,*S. pombe* is an attractive option for synthetic biology applications. However,*S. pombe* currently lacks modular tools for generating genetic circuits with more than 1 transcriptional unit. We developed a toolkit to address this gap. Adapted from the MoClo-YTK plasmid kit for *Saccharomyces cerevisiae* and using the same modular cloning grammar, our POMBOX toolkit is designed to facilitate fast, efficient, and modular construction of genetic circuits in*S. pombe*. It allows for interoperability when working with DNA sequences that are functional in both*S. cerevisiae* and *S. pombe* (e.g., protein tags, antibiotic resistance cassettes, and coding sequences). Moreover, POMBOX enables the modular assembly of multigene pathways and increases the possible pathway length from 6 to 12 transcriptional units. We also adapted the stable integration vector homology arms to Golden Gate assembly and tested the genomic integration success rates depending on different sequence sizes, from 4 to 24 kb. We included 14 *S. pombe* promoters that we characterized using two fluorescent proteins, in both minimally defined (EMM2—Edinburgh minimal media) and complex (YES—yeast extract with supplements) media. Then, we examined the efficacy of 6 *S. cerevisiae* and 6 synthetic terminators in *S. pombe*. Finally, we used the POMBOX kit for a synthetic biology application in metabolic engineering and expressed plant enzymes in *S. pombe* to produce specialized metabolite precursors, namely, methylxanthine, amorpha-4,11-diene, and cinnamic acid from the purine, mevalonate, and aromatic amino acid pathways.

## Introduction

The fission yeast *Schizosaccharomyces pombe* is a well-characterized model organism for molecular and cellular biology of eukaryotes.^1^ As a yeast, *S. pombe* is a unicellular archiascomycete that grows to a high density and is easy to manipulate. Its extensive experimental functional annotations have been compiled in the well-curated database PomBase.^1,2^ These assets make *S. pombe* an attractive organism for synthetic biology where the ideal chassis organism has to be amenable to genetic modification with its behavior precisely predicted and modeled. Among the applications of synthetic biology, metabolic engineering aims to increase the production of high-value chemicals through the modification of organisms that naturally produce these compounds^3^ or by expressing a pathway of interest in a heterologous host^4−6^

Evolutionarily, *S. pombe* diverged from *Saccharomyces cerevisiae* and other yeasts (*Candida*, *Yarrowia*, and *Pichia* spp.) about one billion years ago^7^ and has several characteristics that make it a suitable chassis for metabolic engineering. Basic molecular biology tools, such as reporter genes, the CRISPR/Cas system, and genomic integration, are already available for use with *S. pombe*. Additionally, genome-scale metabolic models have been established for this organism. Unlike *S. cerevisiae*, *S. pombe* retains 4′-phosphopantetheinyl transferase, which is necessary for the synthesis of fungal and bacterial polyketides and nonribosomal peptides,^8^ and produces cofactors required for specific enzymatic reactions, such as vitamin B21. Some projects have proposed using *S. pombe* as a metabolic engineering platform, particularly for overproducing 3-hydroxypropionic acid via the malonyl-CoA pathway,^9^ lactic acid,^10^ ricinoleic acid,^11^ and vanillin,^12^ or for expressing cytochrome P450 with its partner NADPH-cytochrome P450 oxidoreductases (CPRs).^13^ However, these few examples are mostly limited to the expression of a single heterologous enzyme or, at most, in the case of vanillin, a biosynthetic pathway consisting of three enzymes. To recreate the vanillin biosynthetic pathway, Hansen et al. had to sequentially integrate the three genes into three different genomic loci.^12^ The absence of tools for performing metabolic engineering in *S. pombe* has been identified as a critical factor contributing to the lack of nonribosomal peptide production utilizing this organism.^8^

Synthetic biology has greatly contributed to metabolic engineering through the improvement of genome editing^14^ and multigene^15^ assembly tools for the efficient reconstruction and integration of biosynthetic pathways. Promoter and terminator libraries^16,17^ provide finely characterized regulatory elements and minimize construct size and homologous recombination events to ensure the stability and tunable expression of biosynthetic pathways. However, no such tools currently exist for *S. pombe*. For plasmid or integration vector construction, efforts have been made to develop strategies that allow for the modularity of promoter and coding sequences. A toolkit based on Golden Gate assembly, a fast and efficient DNA assembly method to ligate multiple DNA fragments in a single reaction,^18^ was proposed by Kakui et al. in 2015^19^ but only provided three different promoters (*adh1*, *nmt1*, and *urg1*) and a single terminator. Furthermore, this system was not designed for the construction of multiple gene circuits. In 2020, Vještica et al. proposed a series of new integration vectors for *S. pombe*, making the regulatory elements modular.^20^ The stable integration vectors (SIVs) they built allow for efficient and rapid integration of DNA sequences into the *S. pombe* genome. They also characterized six new promoters and used the exogenous terminator tCyc1 from *S. cerevisiae*. Therefore, there is currently no solution for the fast and modular multigene assembly and reconstruction of biosynthetic pathways in *S. pombe*. In 2015, Lee at al.^21^ designed the molecular cloning yeast toolkit (MoClo-YTK) for *S. cerevisiae*. It contains 96 characterized parts split into 8 part types (i.e., connectors left and right, promoter, coding sequence, terminator, yeast marker, origin of replication, and bacterial marker) enabling the streamlined assembly of cassette and multigene plasmids in a modular fashion. Thanks to Golden Gate assembly and its standardized overhangs, this toolkit has been extended for other applications and adapted to other organisms.^22−28^

Here, we introduce POMBOX, a toolkit dedicated to the modular assembly of multigene integration vectors for applications in molecular and synthetic biology. POMBOX reuses the overhangs proposed in the MoClo-YTK^21^ toolkit and is therefore compatible with several other existing molecular biology toolkits, allowing for better interoperability of DNA parts. We also include short synthetic regulatory elements to decrease the size of the constructs and maximize the stability of the constructs after genomic integration.

## Results and Discussion

### Principles of the Toolkit

**Figure 1:**
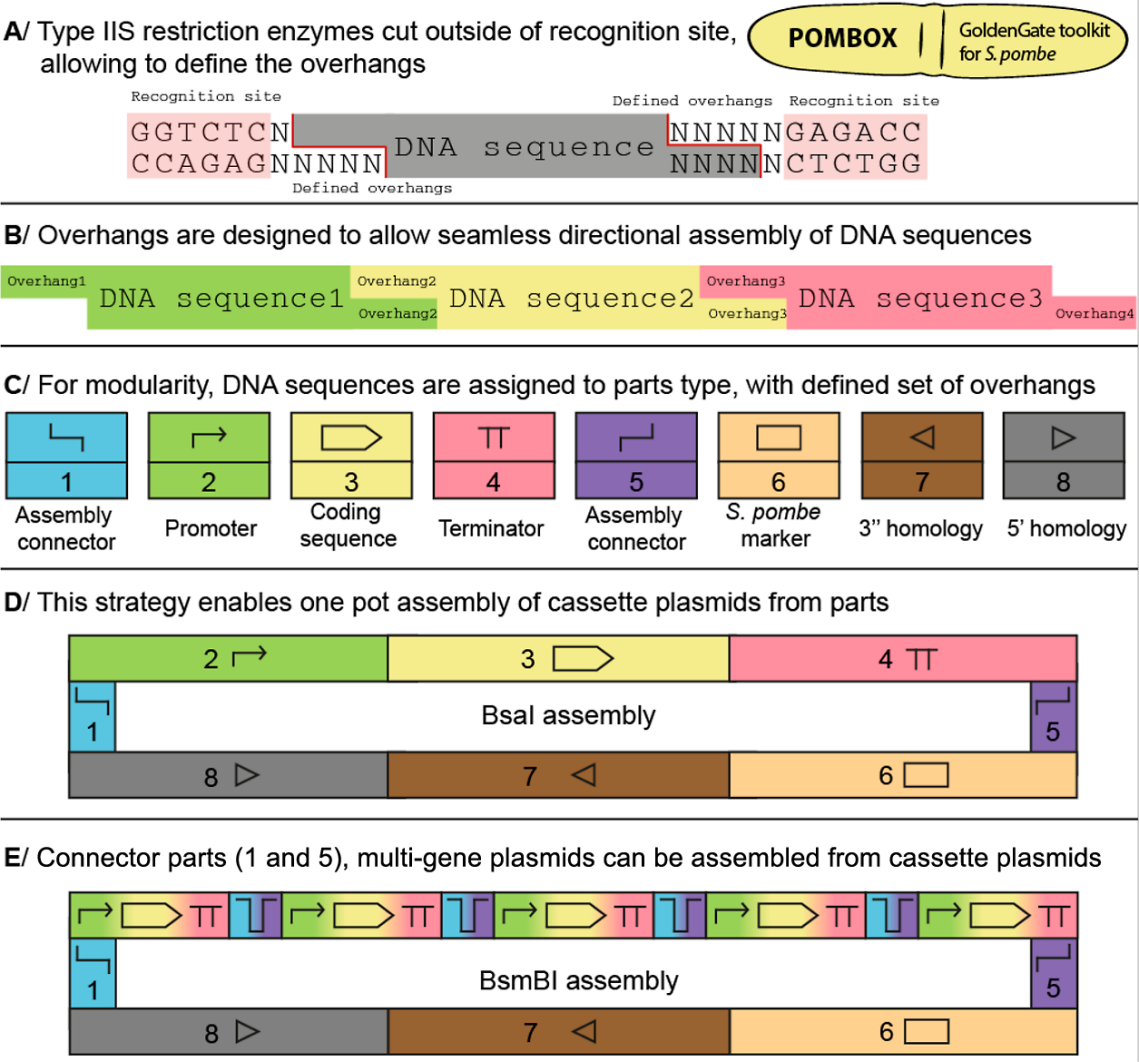
Standardized Golden Gate assembly workflow for plasmid assembly. (A) Golden Gate assembly relies on type IIS restriction enzymes. When the recognition sites are outside the DNA sequence of interest, it allows for seamless assembly. (B) Overhangs can be defined to increase the ligation efficiency and assemble DNA in an ordered fashion. (C) The toolkit relies on 8 part types, each one with a defined set of overhangs. It enables the integration of new compatible parts in the toolkit. (D) Parts are used to assemble, in one pot, a complete plasmid or integration vector using the BsaI enzyme. It enables modularity of sequences and combinatorial assembly. (E) Pairs of connector parts allow for the assembly of multigene plasmids using the BsmBI enzyme.

POMBOX is an extension of MoClo-YTK designed for *S. pombe*. An extensive explanation, details of the workflow, and examples are presented in the Supporting Information and in the original MoClo-YTK article.^21^ Golden Gate assembly is, in principle, based on type IIS restriction enzymes. Unlike classical restriction enzymes, type IIS restriction enzymes cleave DNA outside the recognition site (Figure 1A). The Golden Gate approach offers two significant advantages for molecular biology strategies. First, positioning the recognition sites outside of DNA sequences to be cloned makes it possible to generate products lacking the original restriction site (Figure 1A). Second, as the overhangs can consist of any sequence of four nucleotides, they can be designated upstream to enhance the ligation efficiency,^29,30^ resulting in a MoClo “grammar” that enables reliable ligations (Figure 1B). To harness these properties, the MoClo-YTK toolkit adopts the Golden Gate assembly approach, defining eight types of DNA parts that are essential for generating plasmids or genomic integration vectors. Assembly connectors (parts 1 and 5) facilitate genotyping and multigene assembly. Parts 2, 3, and 4 form the transcription unit, constituted by a promoter (part 2), a coding sequence (part 3), and a terminator (part 4). Additionally, subtypes of parts 3A, 3B, 4A, and 4B have been developed to integrate tags to the coding sequence at the N-terminus or C-terminus (Figure S1). These various parts are then employed in a streamlined one-step Golden Gate reaction (Figure 1D), enabling the assembly of a complete functional plasmid suitable for experimentation or the generation of a multigene plasmid (Figure 1E). To function properly, some core rules have to be followed when generating new parts: (I) DNA sequences should be free of BsaI, BsmBI, and NotI recognition sites, as those three enzymes are used for plasmid assembly and vector linearization and (II) overhangs should respect the MoClo-YTK grammar. Overhangs are listed in the Supporting Information, Tutorial section.

Overall, this molecular biology framework enables fast, efficient, and reliable generation of plasmids. All the backbone vectors used in this toolkit possess a fluorescent protein dropout allowing for fast selection of transformants with a correctly assembled plasmid, thanks to green-white screening. As claimed in the MoClo-YTK paper,^21^ screening one transformant is typically sufficient to find a correctly assembled transformant plasmid. Using POMBOX and the MoClo-YTK assembly principles, we were able to generate up to 24 strains of *S. pombe,* expressing a fluorescent protein under the control of different transcriptional regulators in 7 days.

**Figure 2:**
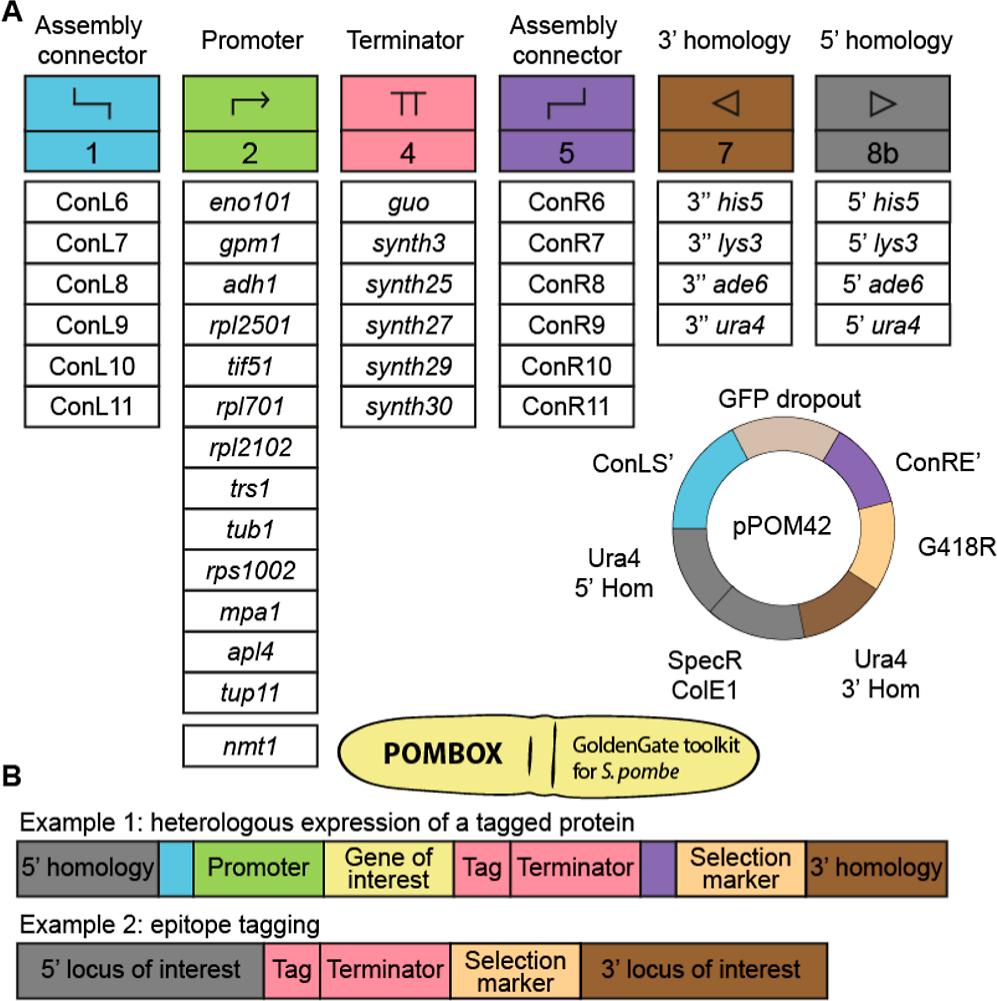
New parts provided by the POMBOX toolkit. (A) The toolkit provides 6 new assembly connector pairs (part type 1 and 5), 14 characterized promoters (part type 2), 6 short synthetic terminator sequences (part type 4), and 4 pairs of homology arms (part type 7 and 8b), domesticated from stable integration vectors.^20^ The POMBOX toolkit also included 2 backbone vectors for genomic integration such a pPOM042 for multigene assembly. (B) Two applications from POMBOX DNA parts. Example 1 highlights a tagged protein overexpression vector and example 2 a vector for epitope tagging.

POMBOX is a collection of characterized DNA parts (Figure 2A), based on the MoClo assembly grammar defined by Lee et al.^21^ We chose this MoClo assembly grammar because some exogenous sequences (antibiotic resistance cassettes, bacterial markers and origins of replication, coding sequences, tags, and assembly connectors; see Figure S1 for a list of compatible parts from the MoClo-YTK toolkit) can be shared between *S. cerevisiae* and *S. pombe*. To complete the toolkit, we propose 40 new parts and 2 integration vectors (pPOM001-042): 6 new pairs of connectors, 14 promoters characterized in two different *S. pombe* culture media, and 2 protein expressions. Then, we verified the compatibility of *S. cerevisiae* terminators and short synthetic terminators in *S. pombe* and adapted the Vještica et al.^20^ strategy for stable genomic integration in *S. pombe* for compatibility with the Golden Gate toolkit. We assessed the impact of the exogenous DNA length on the transformation rate of *S. pombe*. POMBOX DNA parts can be used and adapted for regular molecular biology applications such as protein expression (Figure 1B, example 1), epitope tagging (Figure 2B, example 2), gene deletion, insertion of mutations, and protein–protein interactions. The procedure to generate the two examples of integration vectors from POMBOX is described in the Supporting Information. Finally, to validate the practical utility of POMBOX, we chose metabolic engineering applications and expressed three plant enzymes that generate precursors for the biosynthesis of specialized metabolites in *S. pombe*.

### New Connectors for Multigene Plasmid Assembly

Connector parts enable the generation of multigene plasmids from single transcriptional unit plasmids (e.g., a promoter, a coding sequence, and a terminator). Each pair of connectors features a BsmBI site and a unique overhang for assembling the transcriptional units in a systematic manner. The original YTK toolkit included 6 connector pairs for multigene assembly, but there is a growing demand for larger pathways that can produce specialized metabolites in *S. cerevisiae*,^31^ and the Golden Gate approach has been used to build assemblies of 52 fragments up to 40 kb long.^32^ To meet this demand, we designed 6 new connectors for the construction of larger pathways. The connectors include a 143 bp concatenation of barcode sequences, a BsmBI recognition site, unique overhangs, and a 21 bp barcode scar, as recommended by the MoClo-YTK. To avoid homology with *S. pombe* or *S. cerevisiae* genomes, we selected barcode sequences with no similarity to these genomes.^33^ To ensure that the overhang sequences of the new connectors are accurate, we used the NEBridge Ligase Fidelity platform to achieve assemblies with over 99% fidelity.^29,30^ We named the 6 new connector pairs ConL5-11 and ConR5-11, respectively.

### Collection of Promoters for Protein Expression

**Figure 3:**
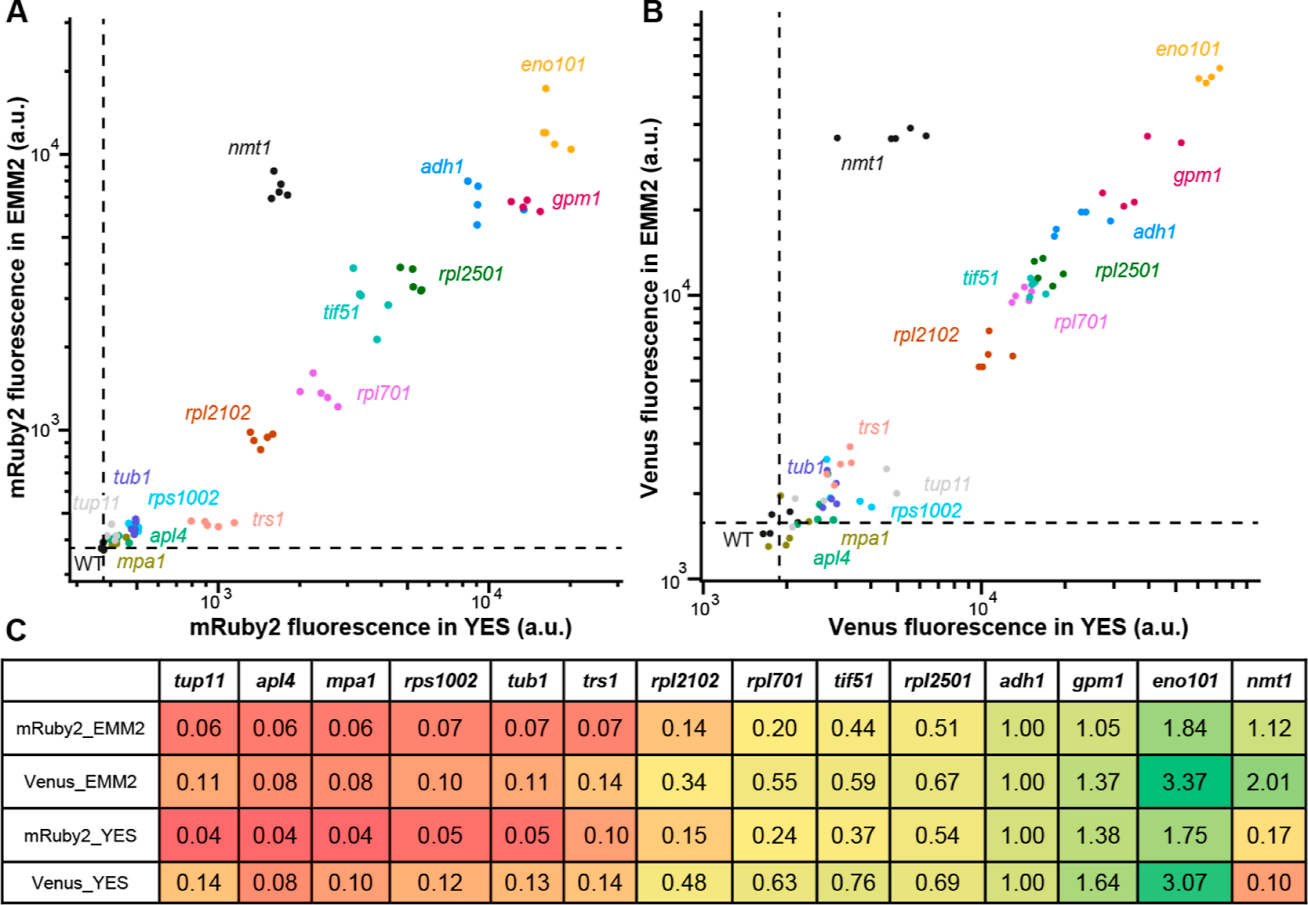
Strength of POMBOX promoters. The strength of 14 promoters was quantified by flow cytometry by measuring the fluorescence emitted by the (A) mRuby2 and (B) venus proteins under the control of each promoter in the EMM2 or YES medium. WT is the background fluorescence from wild-type *S. pombe* cells. (C) Strength of POMBOX promoters was relative to P*adh1*.

We characterized the levels of gene expression for 13 constitutive promoters and 1 regulatable promoter. Three of the 14 characterized promoters (2 constitutive, P*tub1* and P*tif51*, and 1 regulatable, P*nmt1*) are also used in the widely used pDUAL2 vector series.^34^ To cover a wide range of gene expression with POMBOX, we selected 11 additional promoters using transcriptomic data generated by Thodberg et al.^35^ In this study, they analyzed the *S. pombe* transcriptome under two sets of standard culture conditions [EMM2 (Edinburgh minimal media) and YES (yeast extract with supplement)] and three sets of stress-inducing conditions in *S. pombe* cell physiology (YES with 15 min at 39 °C, EMM2 with nitrogen starvation, and YES with 0.5 mM H_2_O_2_ for 15 min).^35^ Thus, promoters showing <5% variability of transcript per million between the five sets of conditions we retained as constitutive. POMBOX selected promoters cover a range of expression levels (expressed as transcripts per million) from 120 to 300,000 (2500-fold change, Figure S2). The promoters were amplified from the genome of *S. pombe* strain *972h–*. They consist of the 5′ UTR and the 1000 bp downstream of the transcription start site. *Ptub1* (also named atb2) and P*tif51* were amplified from the pDUAL2 plasmid series. To evaluate the strength of each promoter, we cloned them upstream of a fluorescent reporter (*mRuby2* or *Venus*)^36,37^ and measured the fluorescence during exponential growth in YES or EMM2 using flow cytometry (Figure 3). Each construct was made using the same terminator (T*eno1*) and integration locus (*ura4*).

The promoters we propose cover 30- to 40-fold fluorescence intensity values and have been tested in two different media, YES and EMM2. These promoters are constitutive, with P*nmt1* as a regulatable promoter, repressed in the presence of thiamine and activated in the absence of thiamine. YES is a complex medium from yeast extract that contains thiamine, whereas EMM2 as a synthetic medium does not. Therefore, P*nmt1* is repressed in the YES and activated in EMM2. For the constitutive promoters, the medium’s composition has no to little impact on the protein expression. Virtually, all *S. pombe* native promoters are compatible with the toolkit and can extend the 14 that we have provided. For interoperability between experiments and laboratories, we also present relative fluorescence values of the 14 promoters normalized to P*adh1* fluorescent protein expression (Figure 3C).

### Collection of Terminators for Protein Expression

**Figure 4:**
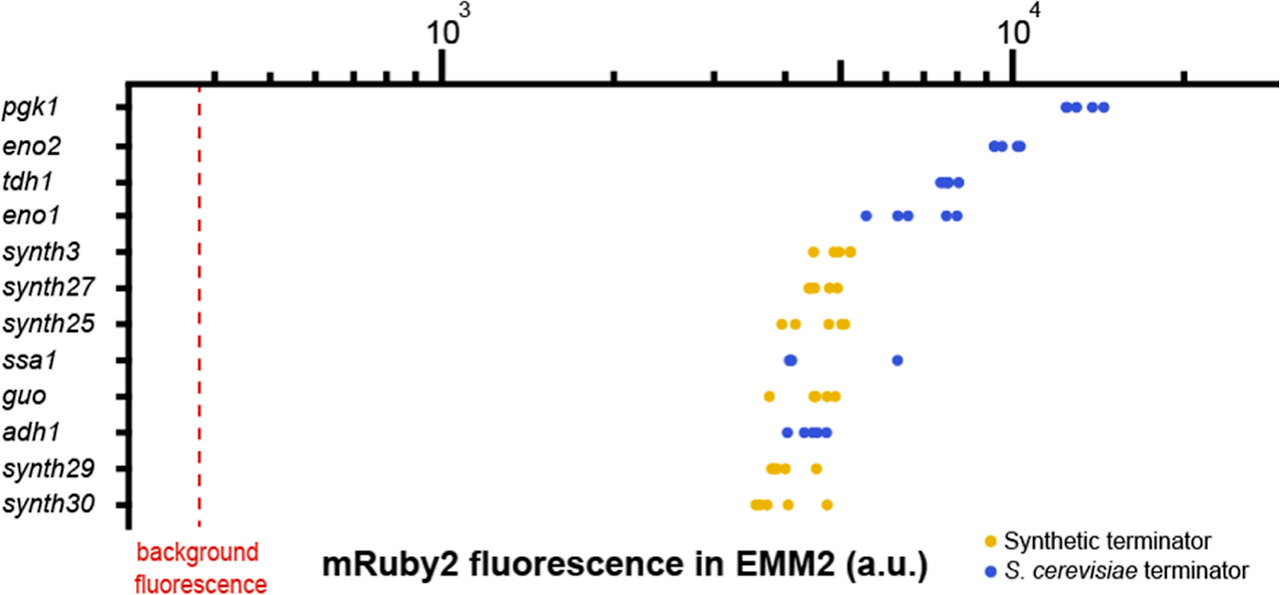
Modulation of protein production by POMBOX terminators. The modulation of protein production by six synthetic terminators and six *S. cerevisiae* terminators from the MoClo-YTK toolkit was quantified by flow cytometry by measuring the fluorescence emitted by the mRuby2 protein under regulation of each promoter in EMM2.

Homologous recombination events after genomic integration caused by sequence homology are undesirable in molecular or synthetic biology applications. The simplest and most effective way to minimize these events, if possible, is to use exogenous sequences that are not similar to genomic sequences. To achieve this, using sequences from evolutionarily distant organisms or synthetic sequences is good practice. It has been shown that transcription terminators can be transferred from *S. cerevisiae* to *Pichia pastoris* while maintaining similar protein production capabilities and that synthetic terminators designed and tested in *S. cerevisiae* also retain their properties in *P. pastoris*.^38^ This has also been shown to be the case empirically in *S. pombe*, since the T*adh1* terminator used in pDUAL vector series, as well as the T*cyc1* terminator used in the Stable Integration Vectors,^20^ were adopted from *S. cerevisiae*. We therefore verified that the six *S. cerevisiae* terminators present in the YTK, including T*adh1*, as well as six short synthetic terminators, can be used in *S. pombe*(39,40) and maintain protein production levels similar to *Tadh1*. To evaluate the protein production level of each terminator, we cloned it downstream of a fluorescent reporter (mRuby2) and measured fluorescence during exponential growth in EMM2 using flow cytometry (Figure 4). Each construct was made using the same promoter P*adh1* and the integration locus *ura4*.

The gene expression difference that we measured between the strongest fluorescence value and the weakest fluorescence value from T*pgk1* and T*synth30* terminators was a 3.4-fold change. All terminators from *S. cerevisiae* provided higher gene expression values than commonly used T*adh1*. Overall, the synthetic terminators displayed lower gene expression values, similar to those of T*adh1* and T*ssa1* from *S. cerevisiae*. On the other hand, the synthetic terminators require a much smaller DNA sequence compared to terminators provided in the yeast toolkit. The synthetic terminators range from 50 to 80 bp, whereas the MoClo-YTK terminators are 230 bp long.

### *S. pombe* as a Chassis for Metabolic Engineering

**Figure 5:**
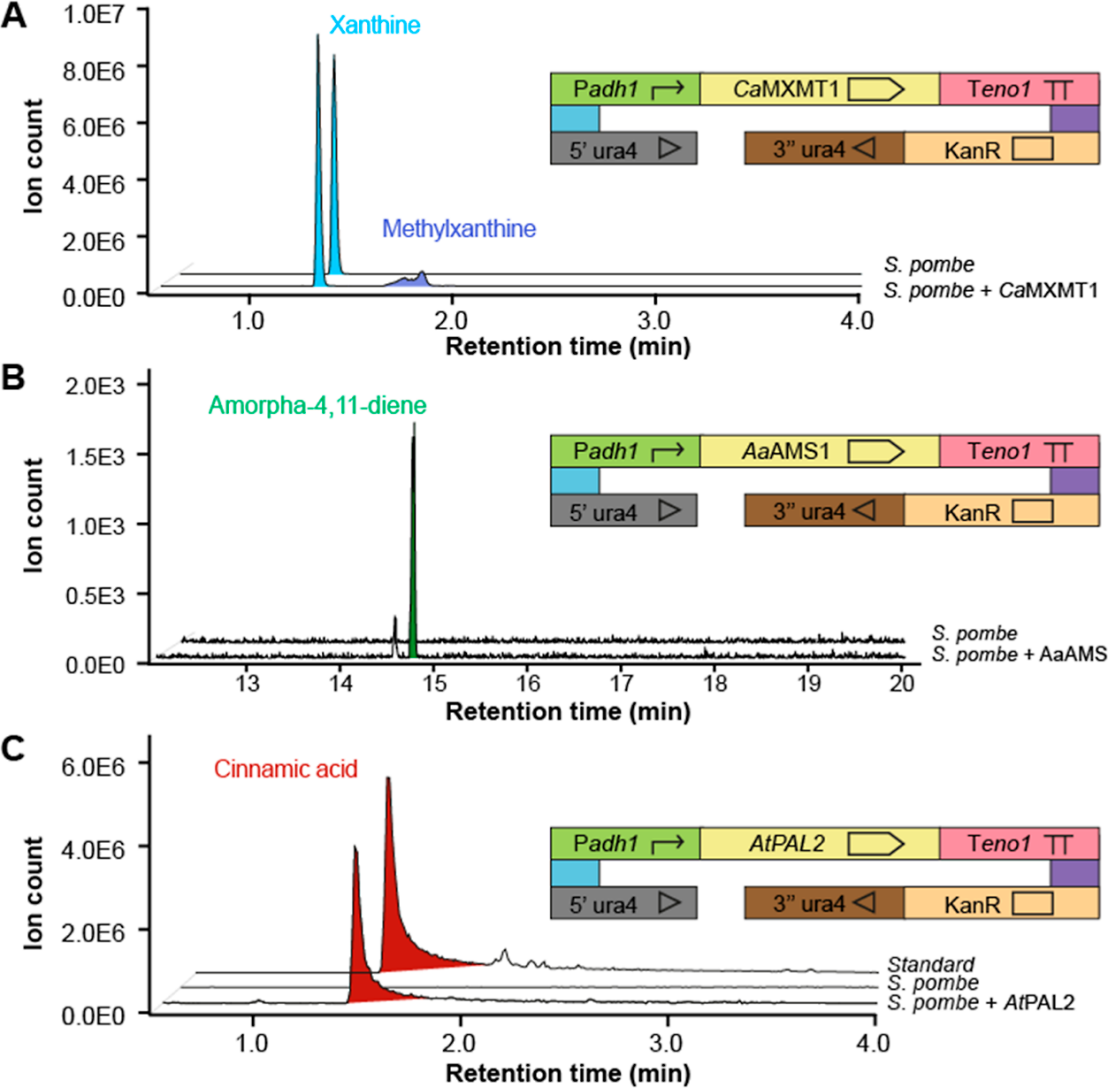
Production of metabolites in a single enzymatic step. (A) Production of methylxanthine from xanthine. (B) Production of amorpha-4,11-diene from farnesyl pyrophosphate. (C) Production of cinnamic acid from phenylalanine.

To emphasize the utility of *S. pombe* as a metabolic engineering chassis, we redirected three metabolic pathways (purine, mevalonate, and aromatic amino acids) to generate precursors of specialized metabolites that are normally not produced in this organism (Figure 5). Monomethylxanthine methyltransferase 1 (MXMT1) from *Coffea arabica* has been described to catalyze the methylation of xanthine, leading to the production of caffeine or theophylline.^41^ We expressed *Ca*MXMT1 under the regulation of P*adh1* and T*eno1*. After analysis of the ethyl acetate extract from *S. pombe* expressing *Ca*MXMT1 using liquid chromatography coupled to positive electrospray ionization high-resolution tandem mass spectrometry (LC–ESI-HRMS/MS), two new peaks with an *m*/*z* of 167.0563 (calculated for [C_6_H_6_N_4_O_2_ + H]^+^, −0.5 ppm) were found in the extracted ion chromatogram (Figure 5A). It shows an effective production of methylxanthines from xanthine by *Ca*MXMT1, supported by MS/MS library matching (Figure S3) and an increase in the retention time. Amorpha-4,11-diene synthase 1 (AMS1) from *Artemisia annua* catalyzes the first reaction toward the production of artemisinin, a sesquiterpenoid with antimalarial properties. We expressed *Aa*AMS1 under the regulation of P*adh1* and T*eno1*. After analysis of the ethyl acetate extract from *S. pombe* expressing *Aa*AMS1 using gas chromatography coupled to electron impact ionization mass spectrometry (GC–EI-MS), a new peak of *m*/*z* 204.2 was found (Figure 5B). The MS spectrum of this compound was matched against the NIST23-EI library, identifying amorpha-4,11-diene as the best match (Figure S4). Phenyl ammonia lyase 2 (PAL2) from *Arabidopsis thaliana* catalyzes the transformation of phenylalanine into cinnamic acid, the first step in the biosynthesis of phenylpropanoids. We expressed *At*PAL2 under the regulation of P*ADH1* and T*eno1*. After analysis of the ethyl acetate extract from *S. pombe* expressing *At*PAL2 using LC–ESI-HRMS/MS, a new peak corresponding to cinnamic acid was found, confirmed by the analysis of a cinnamic acid standard with the same method (Figure 5C).

Thus, using POMBOX, we have successfully integrated into the *S. pombe* genome three different plant enzymes, initiating a specialized metabolite pathway and successfully producing the metabolites of interest.

### Genomic Integration Efficiency of Large Integration Vectors in *S. pombe*

**Figure 6:**
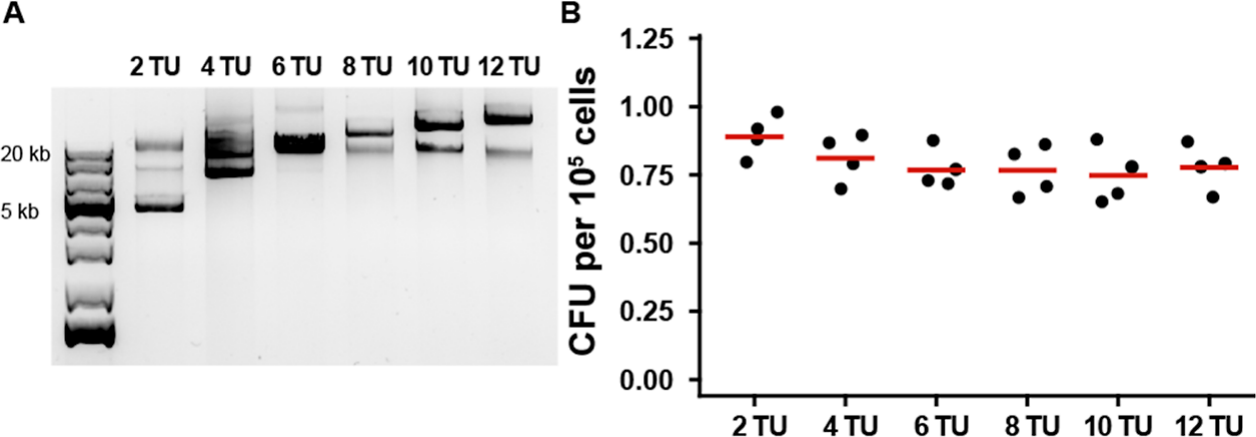
Multigene integration vectors for *S. pombe*. (A) Plasmids from 2 to 12 transcriptional units (TUs) were generated, corresponding to a 4–30 kb sequence to be integrated into the *S. pombe* genome. (B) Transformation efficiency of *S. pombe* as a function of integration vector size, from 6 to 30 kb. CFU: colony-forming units.

Genomic integration by homologous recombination is the most successful and robust method of heterologous expression in *S. pombe*. Recently, Vještica et al.^20^ designed a strategy for stable genomic integration, leading to a reduced false positive transformant rate and consistent integration of a single copy into the targeted locus. We decided to adapt their genomic integration method for POMBOX by removing the BsaI and BsmBI restriction sites from the *ade6*, *his5*, *lys3*, and *ura4* targeting sequences they tested. Then, we wanted to test the genomic integration efficiency of large DNA sequences in *S. pombe*, and we generated multigene plasmids using a combination of MoClo-YTK and POMBOX parts. Those plasmids included 2 to 12 cassette plasmids (Figure 6A). In order to focus solely on the DNA size to be integrated into the genome of *S. pombe*, transcription units were generated by using promoters from *S. cerevisiae*. This was done to prevent the production of RNA or proteins that could hinder the growth of transformed colonies. Purified plasmids were diluted to the exact same number of copies (10^–13^ mol per transformation) and transformed into *S. pombe*. Integration vectors were targeted at the *ura4* locus. DNA sequences up to 24 kb were successfully integrated into the *S. pombe* genome with a transformation efficiency range of 0.895–0.768 colony-forming units (CFU) per 10^5^ cells. These values are on the same order of magnitude as previously reported in the study describing the stable integration vectors.^20^ We observed a slight reduction in the transformation efficiency depending on the size of the DNA sequence.

## Conclusions

To support the adoption of *S. pombe*, a model organism in molecular biology and cell physiology, as a chassis for synthetic biology, we developed POMBOX, a collection of parts compatible with the MoClo-YTK toolkit for the hierarchical Golden Gate assembly. As a first step toward the use of *S. pombe* as a chassis organism in synthetic biology, we characterized 14 promoters and 6 exogenous and 6 synthetic terminators, expressed three plant enzymes that successfully produced precursors of high-value metabolites, and constructed and integrated multipart plasmids up to 12 parts and 30 kb.

## Materials and Methods

### Growth Media and Strains

Chemicals used for media preparation were purchased from either Sigma-Aldrich, Duchefa Biochemie (Haarlem, Netherlands), Lach:ner (Neratovice, Czech Republic), or Penta Chemicals (Prague, Czech Republic). Solvents for metabolic sample preparation, LC–MS, and GC–MS were purchased from Fisher Chemical and were of LC–MS grade. EMM2 medium was prepared according to Petersen & Russell^42^ with ammonium chloride, 5 g·L^–1^; potassium hydrogen phthalate, 3 g·L^–1^; Na_2_HPO_4_, 2.2 g·L^–1^; glucose, 20 g·L^–1^; salt stock, 50×; vitamin stock, 1000×; and mineral stock, 10,000×. YES medium was prepared according to Petersen & Russell^42^ with 30 g·L^–1^ glucose, 5 g·L^–1^ yeast extract, 0.2 g·L^–1^ adenine, 0.2 g·L^–1^ uracil, 0.2 g·L^–1^ histidine, 0.2 g·L^–1^ leucine, and 0.2 g·L^–1^ lysine. For solid media, 20 g·L^–1^ of agar was added.

*S. pombe* wild-type strains *h– 972* and *h+ 975 ura4*-D18 were used as the starting strains. *S. pombe972h–* was used as a template for all DNA part amplifications. *S. pombeh+ 975 ura4-D18* was used as a starting strain for genetic engineering. The complete list of strains generated for this study is available in Table S6.

DH10 electrocompetent *E. coli* cells were used for all molecular cloning experiments. Transformed cells were selected on lysogeny broth (LB) with appropriate antibiotics (ampicillin, chloramphenicol, or spectinomycin). SOC media was used for recovery after electroporation.

### Growth Conditions

For maintenance, *S. pombe* strains were cultivated in YES or EMM2 solid media at 28 °C.

For general preculture, *S. pombe* strains were cultivated in YES or EMM2 at 28 °C and 200 rpm in an orbital shaker.

For experiments measuring the promoter and terminator strength, *S. pombe* strains were cultivated in 1 mL volumes in 96-deep-well plates (CR1496, Enzyscreen) sealed with AeraSeal (Excel Scientific), at 28 °C and 1500 rpm on an Eppendorf ThermoMixer C (Eppendorf).

For metabolic pathway expression experiments, *S. pombe* strains were cultivated in EMM2 in 24-deep-well plates (CR1426, Enzyscreen) sealed with AeraSeal (Excel Scientific), at 28 °C and 800 rpm on an Eppendorf ThermoMixer C (Eppendorf).

For plasmid amplification, *E. coli* strains were cultivated in LB medium in 24-deep-well plates (CR1426, Enzyscreen) sealed with AeraSeal (Excel Scientific), at 37 °C and 800 rpm on an Eppendorf ThermoMixer C (Eppendorf).

### Plasmids

The list of all plasmids used in the study is presented in Tables S1–S5.

All pPOM and pTP plasmids were generated using Golden Gate assembly based on the toolkit and overhangs of Lee et al.^21^

Part plasmids (Tables S1–S3) used pYTK001 as a backbone. DNA parts were either synthesized by TwistBioscience as gene fragments (*Aa*AMS1, *Ca*MXMT1, ConL6-11, and ConR6-11) or integration vectors (3′His5 and 5′Lys3), GenScript subcloned in PUC57-BsaI-BsmBI-free (T*guo*, T*synth3*, T*synth25*, T*synth27*, T*synth29*, and T*synth30*) or amplified from the *S. pombe* 972h- genome, pDUAL-FFH21 (P*tub1*), and pDUAL-FFH51 (P*tif51*) using overhang PCR. In some cases (P*nmt1*, P*adh1*, 5′-ura4, 5′-ade6, 3′-lys3, 5′-lys3, and 3′-his5), a mutation was inserted using overlap extension PCR to remove BsmBI or BsaI restriction sites.

Cassette plasmids (Table S4) used pPOM041 or pYTK095 as the backbone. pPOM41 was used for direct integration of cassettes into the *S. pombe* genome and pYTK095 for multigene plasmid assembly. Multigene plasmids (Table S5) used pPOM042 as the backbone.

Plasmid extraction was achieved from 2 mL of LB of an *E. coli* plasmid-harboring overnight culture, using a QIACube robot (Qiagen) and the QIAprep Spin Miniprep Kit (27104, Qiagen), following the QIAprep miniprep protocol, either the “rapid” option for plasmids <10 kb or the “plasmid 10 kb or larger” option for plasmids >10 kb. Plasmids were eluted in 50 μL of ddH_2_O.

### Polymerase Chain Reaction

For the amplification of DNA parts from *S. pombe* or plasmids and overlap extension PCR, we used Phusion High-Fidelity DNA Polymerase (M0530L, New England Biolabs) under the following conditions:

In the final 20 μL are 4 μL of GC buffer, 0.4 μL of 10 mM dNTPs, 1 μL of 10 μM forward primer, 1 μL of 10 μM reverse primer, 1 μL of DNA template, 0.2 μL of Phusion polymerase, and 12.6 μL of ddH_2_O. Reactions were conducted in a ProFlex 3 × 32-well PCR system thermocycler (Applied Biosystem). Thermocycling conditions used the following template: 98 °C for 5 min as initial denaturation; for 35 cycles: 98 °C for 15 s, annealing temperature for 15 s, 72 °C for 30 s·kb^–1^, and final extension at 72 °C for 5 min. PCR products were separated on agarose gels (1% w/v), 90 V, 60 min, and purified using NucleoSpin Gel and PCR Cleanup (Macherey-Nagel) before subsequent use.

For colony PCR and yeast genotyping, we used the Phire Green Hot Start II PCR Master Mix (Thermo Fisher Scientific) under the following conditions:

In the 10 μL final are 5 μL of Phire Green Hot Start II PCR Master Mix, 0.2 μL of 25 μM forward primer, 0.2 μL of 25 μM reverse primer, and 4.6 μL of DNA template. Reactions were conducted in a ProFlex 3 × 32-well PCR system thermocycler (Applied Biosystem). Thermocycling conditions used the following program: 98 °C for 2 min as initial denaturation; for 30 cycles: 98 °C for 10 s, annealing temperature for 10 s, 72 °C for 10 s·kb^–1^, and a final extension 72 °C for 2 min. PCR products were separated on an agarose gel (0.8% w/v), 130 V, 30 min.

*E. coli* colonies were selected with a toothpick and spotted 4 times on selective media. The remaining bacteria were then resuspended in 10 μL of ddH_2_O and boiled for 10 min before being used as a DNA template.

Yeast genotyping was adapted from Lõoke et al.;^43^*S. pombe* colonies were selected with a toothpick and resuspended in 100 μL of 200 mM LiOAc and 1% SDS solution and boiled for 10 min. Then, 300 μL of 96% EtOH was added, and the solution was vortexed and centrifuged at 15,000*g* for 3 min. The supernatant was discarded, and the pellet was washed with 500 μL of 70% EtOH before centrifugation at 15,000*g* for 1 min. The supernatant was discarded, and the pellet was dried for 1 min at room temperature. The precipitated DNA was dissolved in 100 μL ddH_2_O, and cell debris was spun down at 15,000*g* for 1 min.

### Golden Gate Assembly Reaction

Part plasmids were generated in a 10 μL reaction volume—T4 ligase buffer, 1 μL; T4 ligase, 0.5 μL (M0202L, New England Biolabs, Ipswich, Massachusetts, United States); BsmBI-v2, 0.5 μL (R0739L, New England Biolabs, Ipswich, Massachusetts, United States); pYTK001, 0.5 μL; DNA part, 0.5 μL (20 fmol); and ddH_2_O, up to 10 μL. Reactions were conducted in a ProFlex 3 × 32-well PCR system thermocycler (Applied Biosystem, Waltham, Massachusetts, United States). Thermocycling conditions used the following program: for 25 cycles, 42 °C for 2 min, 16 °C for 2 min, and then 60 °C for 30 min and 80 °C for 10 min.

Cassette plasmids were generated in a 10 μL reaction volume—T4 ligase buffer, 1 μL; T4 ligase, 0.5 μL (M0202L, New England Biolabs, Ipswich, Massachusetts, United States); BsaI-HFv2, 0.5 μL (R3733L, New England Biolabs, Ipswich, Massachusetts, United States); DNA parts, 0.5 μL (20 fmol); and ddH_2_O, up to 10 μL. Reactions were conducted in a ProFlex 3 × 32-well PCR system thermocycler (Applied Biosystem, Waltham, Massachusetts, United States). Thermocycling conditions used the following program: for 25 cycles, 37 °C for 5 min, 16 °C for 5 min, then 60 °C for 30 min, and 80 °C for 10 min.

For backbone plasmid assembly, thermocycling conditions were modified to end after a long ligation step: for 25 cycles, 42 °C for 5 min, 16 °C for 5 min, and then 16 °C for 30 min.

Multigene plasmids were generated in 10 μL reaction volume—T4 ligase buffer, 1 μL; T4 ligase, 0.5 μL (M0202L, New England Biolabs); BsmBI-v2, 0.5 μL (R3733L, New England Biolabs); DNA parts, 0.5 μL (20 fmol); and ddH_2_O, up to 10 μL. Reactions were conducted in a ProFlex 3 × 32-well PCR system thermocycler (Applied Biosystem). Thermocycling conditions used the following template: for 25 cycles, 42 °C for 5 min, 16 °C for 5 min, then 60 °C for 30 min and 80 °C for 10 min.

### *E. coli* Transformation

Electroporation cuvettes were cooled to 4 °C for 30 min before each experiment. A 20 μL volume of a suspension containing electrocompetent *E. coli* cells was thawed at 4 °C for 10 min before each experiment. Once thawed, 0.5 μL of plasmid was added to the cells with gentle mixing. The electroporator was set to 1700 V. For chloramphenicol, spectinomycin, and kanamycin selective markers, cells recovered in 1 mL of SOC for 1 h at 37 °C and 200 rpm. Thus, cells were concentrated to 100 μL through centrifugation at 5000*g* for 3 min and plated to their respective LB + selection marker. In the case of ampicillin, cells were resuspended in 100 μL of SOC after electroporation and directly plated in LB + ampicillin. The cells were then grown overnight at 37 °C.

### *S. pombe* Transformation

Fission yeast strains were generated by the standard lithium acetate transformation protocol^44^ and selected using auxotrophy.

A total of 500–1500 ng of plasmid was digested using NotI-HF in 8 μL of final volume. Cells were precultured overnight in YES medium, 28 °C, 200 rpm. Then, cells were diluted to OD 0.1 and cultivated in YES medium, 28 °C, 200 rpm until they reached OD 0.5. For 5 mL of cells at OD 0.5, the cells were pelleted at 2500*g* for 5 min and washed in 5 mL of sterile ddH_2_O. The cells were pelleted again at 2500*g* for 5 min and resuspended in 1 mL sterile ddH_2_O, pelleted again at 16,000*g* for 1 min, and resuspended in 1 mL TE/LiAc. Then, cells were concentrated in 100 μL of TE/LiAc, then 8 μL of digested plasmid and 10 μL of Salmon sperm DNA were added, mixed gently, and incubated for 10 min at room temperature. Then, 260 μL of TE/LiAc/PEG (40% w/v) was added, and the cells were incubated for 1 h at 30 °C. 43 μL of DMSO was added, and the cells were heat-shocked at 42 °C for 5 min, centrifuged at 6000*g* for 1 min, and washed in water before plating on the EMM2 medium. The cells were grown for 3–5 days at 28 °C.

### Flow Cytometry

*S. pombeh+ 975 ura4*-D18 were maintained in solid EMM2 media and their preculture in 1 mL EMM2 media in 96-deep-well plates (CR1496, Enzyscreen) sealed with AeraSeal (Excel Scientific), at 28 °C and 1500 rpm on an Eppendorf ThermoMixer C (Eppendorf) for 1 day. The cells were seeded at OD 0.05 in 1 mL of EMM2 media in 96-deep-well plates and cultivated for 16 h at 28 °C and 1500 rpm. The cells were then washed in PBS and diluted to OD1 in PBS before analysis by flow cytometry.

Flow cytometry experiments were performed on a CytoFLEX LX flow cytometer (Beckman Coulter). The following channels were used: B525-FITC (525/40 nm filter) and Y610-mCherry (610/20 nm filter). At least, 15,000 events were acquired from singlet-gated populations using FSC-A/SSC-A.

Data were processed using the FlowJo software package with the following gating strategy: (1) the main cellular population was selected using forward and side scatter to exclude cell aggregates and debris and (2) doublets were excluded from analysis by plotting FSC-A versus FSC-H and gating along the diagonal. The number of cells and population mean were extracted to a.csv file. The data were then analyzed using the ggplot2 package in R. Each set of conditions was tested five times in independent experiments.

### Metabolite Extraction

*S. pombeh+ 975 ura4*-D18 cells, with or without genetic modification, were maintained on solid EMM2 media and precultured in 1 mL of EMM2 media in 96-deep-well plates (CR1496, Enzyscreen) sealed with AeraSeal (Excel Scientific), at 28 °C and 1500 rpm on an Eppendorf ThermoMixer C (Eppendorf) for 1 day. Cells were seeded at OD 0.05 in 2 mL EMM2 media in EMM2 in 24-deep-well plates (CR1426, Enzyscreen) sealed with AeraSeal (Excel Scientific, Victorville, California), at 28 °C and 300 rpm for 5 days.

Then, 1 mL of ethyl acetate (E196-4, Fisher Scientific) was added to the culture medium and mixed for 1 h at 28 °C and 250 rpm. Subsequently, 1 mL of ethyl acetate was added and thoroughly mixed by pipetting. The sample was collected in a 2 mL round-bottom tube (Eppendorf), centrifuged for 5 min at 14,100*g*, and the organic phase was carefully collected and dried under a N_2_ flow.

### LC–MS/MS

For LC–MS/MS analysis, samples were resuspended to 1 mg·mL^–1^ in an ACN/H_2_O (50:50) mixture. LC–MS analyses were performed using a Vanquish Flex UHPLC System interfaced to an Orbitrap ID-X Tribrid mass spectrometer equipped with a heated electrospray ionization (H-ESI) source. The LC conditions were as follows: column—Waters BEH (Ethylene-Bridged Hybrid) C18, 50 × 2.1 mm, 1.7 μm; mobile phase—(A) water with 0.1% formic acid and (B) acetonitrile with 0.1% formic acid; flow rate—350 μL·min^–1^; column oven temperature—40 °C; injection volume—1 μL; linear gradient of 5 to 100% B over 5 min and isocratic at 100% B for 2 min. ESI was achieved in positive mode, and mass spectrometer parameters were as follows: ion transfer tube temperature, 325 °C; auxiliary gas flow rate, 10 L·min^–1^; vaporizer temperature, 350 °C; sheath gas flow rate, 50 L·min^–1^; capillary voltage, 3000 V; MS resolution, 60,000, quadrupole isolation; scan range, *m*/*z* 100–1000; RF Lens, 45%; and maximum injection time, 118 ms. The data-dependent MS/MS events were acquired for the most intense ions from the MS scan for a cycle time of 0.6 s, above a threshold of 1.0 × 10^5^ intensity threshold, with a dynamic exclusion list of 2 s, including the isotopes. Selected precursor ions were fragmented with a fixed normalized HCD collision energy of 35%, an isolation window of *m*/*z* 0.8, and a resolution of 15,000 with a maximum injection time of 80 ms.

### GC–MS

For GC–MS analysis, 1 mg samples were resuspended in 100 μL of ethyl acetate.

GC–MS analyses were performed using a 7890A gas chromatograph coupled with a 5975C mass spectrometer, equipped with an EI source and a quadrupole analyzer (Agilent Technologies). The samples (1 μL) were injected into the split/splitless inlet in split mode (split ratio 10:1). The injector temperature was 250 °C. A DB-1 ms fused silica capillary column (30 m × 250 μm; film thickness of 0.25 μm, J&W Scientific) was used for separation. The carrier gas was helium at a constant flow rate of 1.0 mL·min^–1^. The temperature program was 40 °C (1 min), then 5 °C·min^–1^ to 100 °C, followed by 15 °C·min^–1^ to 230 °C. The temperatures of the transfer line, ion source, and quadrupole were 320, 230, and 150 °C, respectively. EI spectra (70 eV) were recorded from *m*/*z* 25 to 500.

### Data Analysis

LC–MS/MS .raw data files were directly imported into MZmine 3.4.16.^45^ Extracted ion chromatograms for compounds of interest were generated using the Raw data overview module and exported as .pdf.

GC–MS .dx files were analyzed using OpenLab CDS 2.4. Extracted ion chromatograms for compounds of interest were exported as .csv files and plotted using the ggplot2 package in R.

Figures were generated using RStudio and the following packages: ggplot2, gridExtra, patchwork, dplyr, forcats, ggthemes, ggprism, DescTools, tidyverse, and scales. Adobe Illustrator CS6 was used for graphic adjustment.

## Data Availability

Information to obtain the POMBOX plasmid kit or individual plasmids is available at https://pluskal-lab.github.io/pombox/.

## Supporting Information Available

Plasmids_POMBOX and tables (ZIP)
Lists of plasmids, strains, and primers; MoClo-YTK parts that can be used with *S. pombe*; promoter strength in transcripts per million; MS/MS spectral comparison of methylxanthine; EI-MS spectrum comparison of amorpha-4,11-diene; and tutorial on how to use POMBOX (PDF)
