# Supplementary material for: POMBOX: A Fission Yeast Cloning Toolkit for Molecular and Synthetic Biology
Source: ACS Synth Biol. 2023 Nov 22;13(2):558–67. doi: 10.1021/acssynbio.3c00529 (PMC10877588; doi:10.1021/acssynbio.3c00529)
Supplement: Supplementary file 2 — sb3c00529_si_002.pdf [file sb3c00529_si_002.pdf]

Supplementary Information

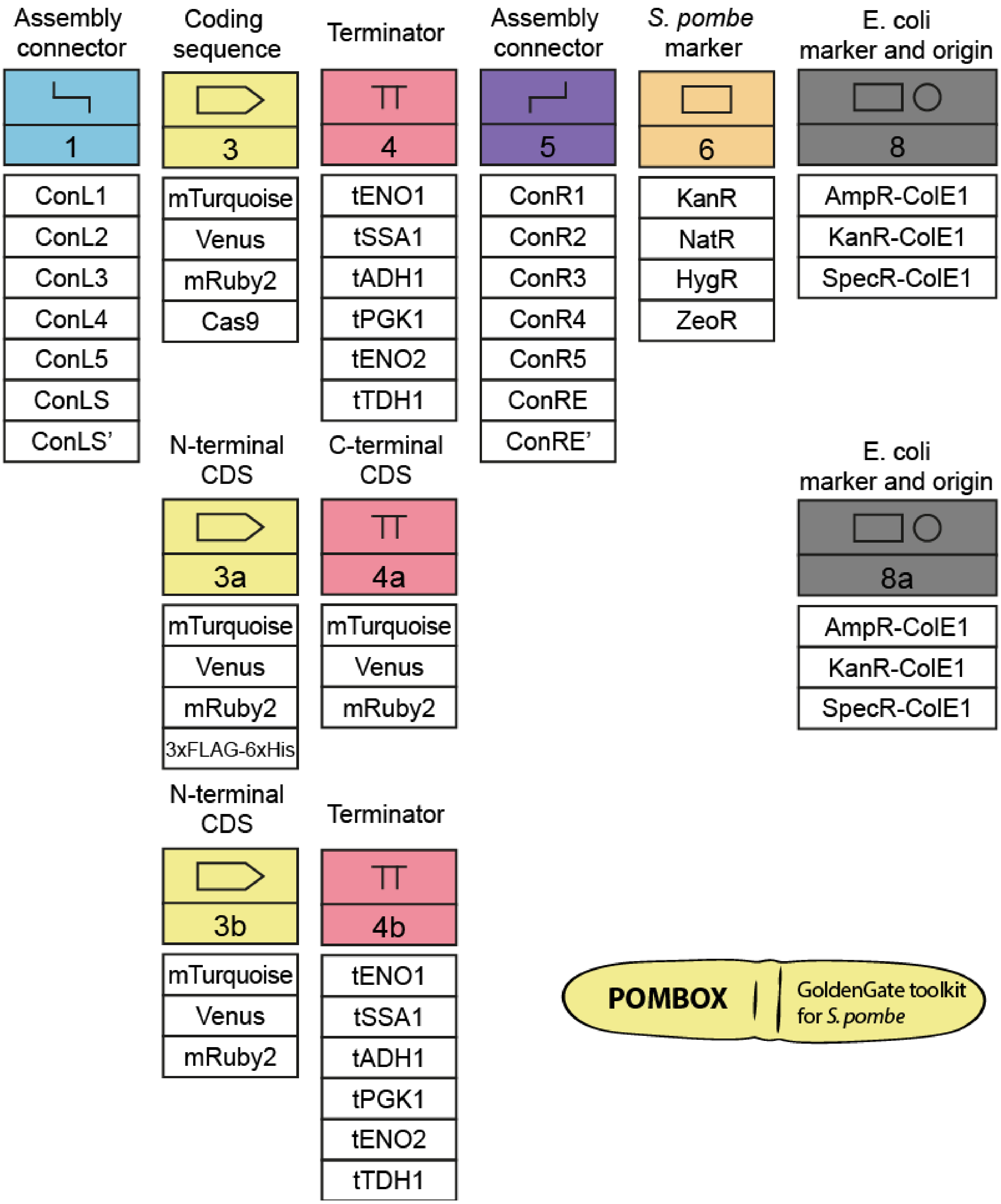

Figure S1: The parts from the original MoClo-YTK kit that can be used with *S. pombe*.

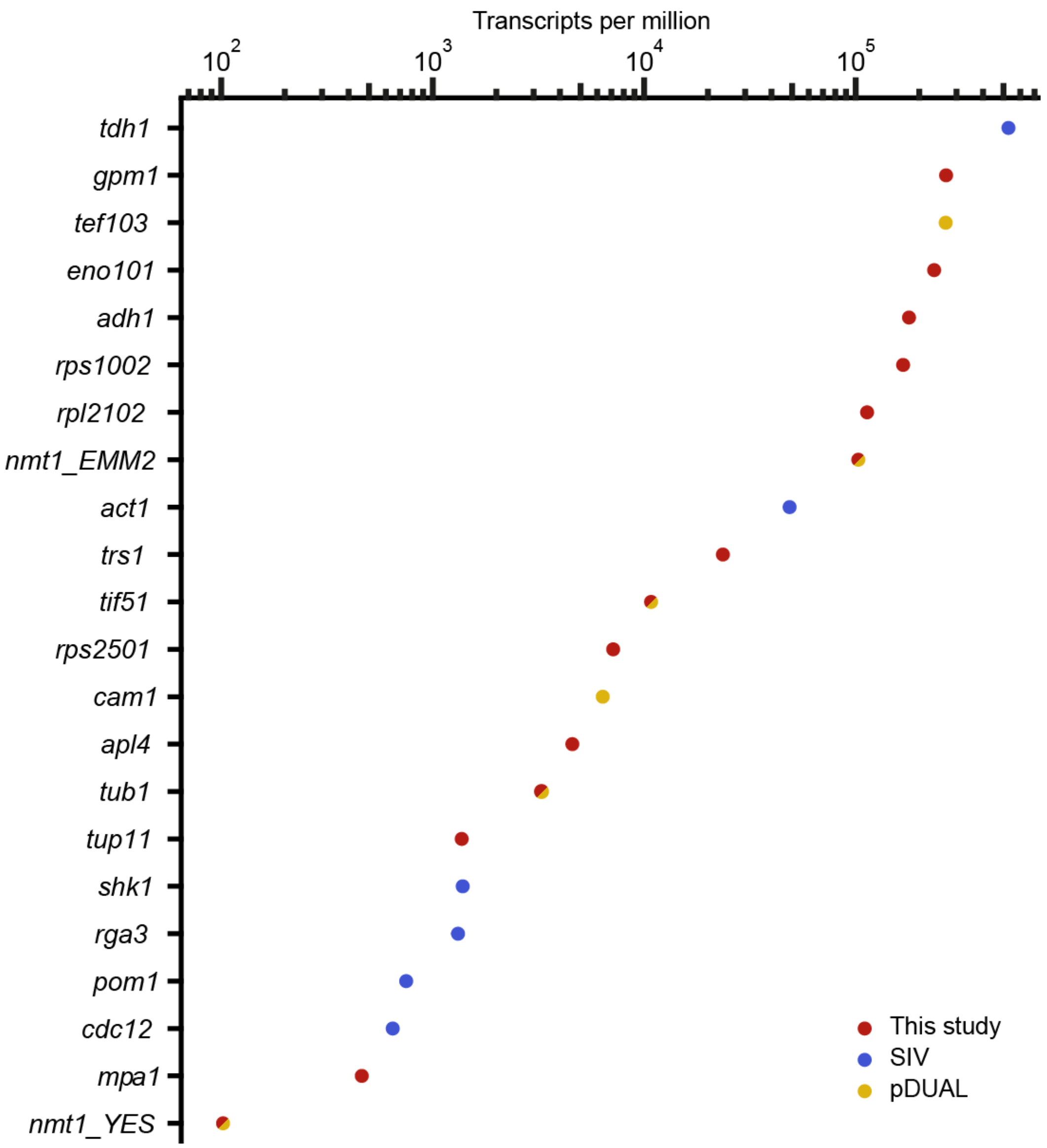

Figure S2: Promoters strength in transcript per million (TPM), measured by Thodberg et al.[35] Red, selected for this study, blue, from Stable Integration Vectors tested by Vjestica et al.,[20] yellow, in the pDUAL2 series.

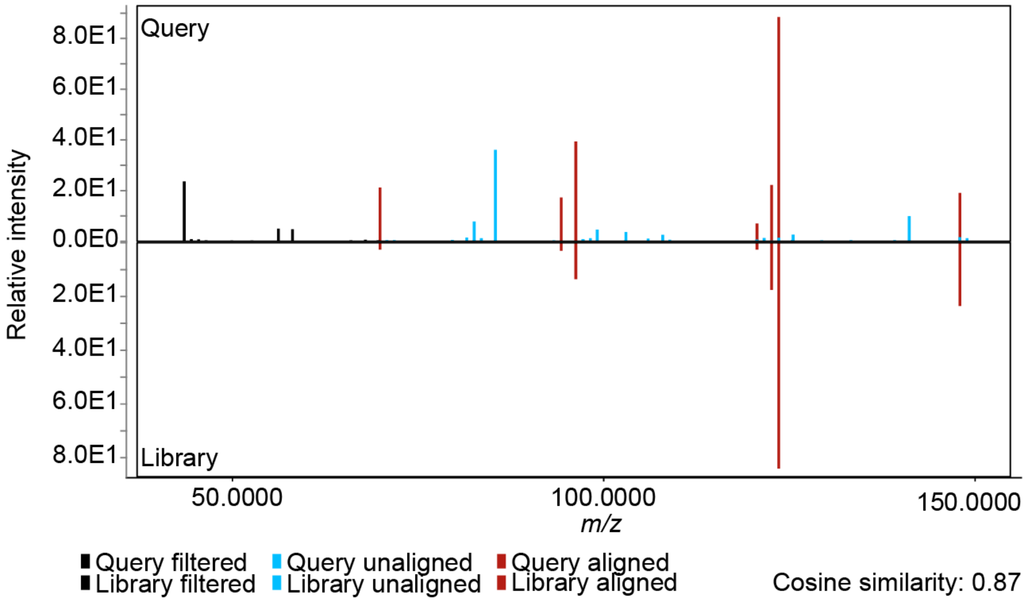

Figure S3: MS/MS spectral comparison of methylxanthine produced by *S. pombe*_CaMXMT1 with 3-methylxanthine library match (Library ID: MSBNK-RIKEN_ReSpect-PT110310).

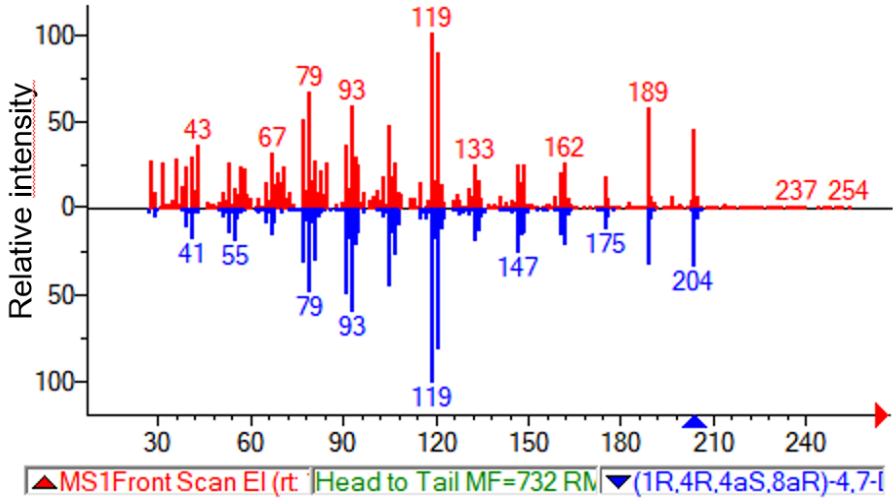

Figure S4: EI–MS spectrum comparison of amorpha-4,11-diene produced by *S. pombe*_AaAMS1 and amorpha-4,11-diene from NIST EI library. Screenshot from NIST MS search 3.0 software.

Tutorial on how to use POMBOX

To support the use of POMBOX, we present two illustrative examples of how to work with Golden Gate assembly and design new parts for molecular biology experiments.

Example 1 is the most straightforward usage of POMBOX. It showcases a method of tagged protein overexpression targeting a classical prototrophy locus. It shows how to make a DNA sequence of interest compatible with POMBOX grammar and describes the experimental workflow.

Example 1 illustrates how to domesticate DNA parts for use in Golden Gate assembly.

Example 2 mimics the usage of pFA plasmids for epitope tagging. It aims to illustrate the versatility and modularity of Golden Gate assembly in molecular biology applications with more advanced design of sequences and choices of overhangs.

Example 2 illustrates the versatility of Golden Gate assembly and how to tweak the overhang grammar for any molecular biology application.

For an in-depth understanding of the design of part types, we recommend reading the supplementary information associated with the MoClo-YTK article.[21]

Example 1: heterologous expression of a tagged protein in S. pombe*.*

For this example, we will consider Cox4 and Cox5, which are two proteins whose structure has recently been resolved using epitope tagging in *S. pombe*[46]*.* Our goal is to overproduce proteins independently and to purify them with the help of the 6XHis tag.

Parts already proposed in POMBOX and MoClo-YTK.

With POMBOX and MoClo-YTK, some parts are already available and ready to use:

- **pPOM041** is the backbone plasmid for genomic integration. It holds parts 1, 5, 6, 7 and 8. It targets the *ura4* locus and brings KanR as a selection marker. pPOM041 also provides a GFP drop-out system for green-white colony screening.
- **pPOM013**: with P*eno101* is selected as part type 2. It is the strongest promoter available.
- **pYTK060**: with the 3XFLAG-6XHis tag
- **pYTK064**: with T*pgk1* is selected as part type 4b (terminators compatible with C-ter tags).

New DNA parts have to be generated.

For this specific study, two coding sequences (part type 3) have to be generated. Those parts are the coding sequence for the Cox4 and Cox5 proteins. There are two methods to obtain those parts:

A/ The coding sequences are synthesized as gene fragments.

B/ The sequences are obtained by amplification from the source organism.

Whatever the chosen approach, both sequences have to respect some core rules to be compatible with POMBOX and MoClo-YTK.

- The DNA sequence should be free of BsaI, BsmBI and NotI recognition sites.
- The overhangs should be part type 3 overhangs. See the list of overhangs at the end of the tutorial section for the exhaustive list.

For method A, gene fragment synthesis, we recommend designing sequences in the following way:

```
Flanking CGTCTCatcGGTCTCaTATG DNASEQUENCE ATCCtGAGACCtGAGACG Flanking
         ______   ______ ____             ____ ______ ______
         BsmBI    BsaI   Overhang     Overhang BsaI   BsmBI
```

The sequence should include the coding sequence of interest (DNASEQUENCE, in this case Cox4 or Cox5), the **type 3 part overhangs**, the **BsaI recognition site** and the **BsmBI recognition site**. We also recommend adding some **flanking** nucleotides to facilitate the binding of the restriction enzymes.

Note that type 3 part overhangs already include the **start codon**.

For method B, amplification of the sequence, we recommend proceeding as follows:

- 1/ Check for any BsaI, BsmBI and NotI restriction sites in your sequence of interest. In the case of Cox4, there are none.

In the case of Cox5, there is one at position 376–381.

- 2a/ For the Cox4 sequence: DNA can be amplified by overhang PCR directly from the *S. pombe* genome. The set of PCR primers can be the following:

```
Forward:   actagacaacCGTCTCatcGGTCTCaTATGTATCTTTCAAAAATTATCTGCAAAAAAGTGCC
Reverse:   acaacacaacCGTCTCaGGTCTCaGGATTTATTTCTTGTCCTTTTCGGAGGGG
```

Note: We recommend using touch-down PCR and gel clean-up to obtain the DNA fragment of interest.

- 2b/ For the Cox5 sequence: DNA can be amplified directly from the genome using overhang and overlap PCR. The two primers can be the following:

```
Forward1:  actagacaacCGTCTCatcGGTCTCaTATGTATCTTTCAAAAATTATCTGCAAAAAAGTGCC
Reverse1:  CGGATCAGACCAAACAATACGAC
Forward2:  GTCGTATTGTTTGGTCTGATCCG
Reverse2:  acaacacaacCGTCTCaGGTCTCaGGATTTATTTCTTGTCCTTTTCGGAGGGG
```

Note: The mutation is underlined. We recommend using touch-down PCR and gel clean-up to obtain the DNA fragment of interest.

- 3/ Store the DNA fragment in pYTK001. It uses BsmBI Golden Gate assembly, and pYTK001 possesses a GFP drop-out for green/white screen of transformant.
  If the DNA fragment was synthesized, we perform BsmBI Golden Gate assembly with a molar ratio of 1:1 of pYTK001 and the DNA fragment, 20 fmol.
  If the DNA fragment was obtained by amplification, we perform BsmBI Golden Gate assembly with 20 fmol of pYTK001 and 1 µL of the DNA fragment from gel clean up.
  We then use 0.5 µL of the Golden Gate reaction mix for 20 µL of competent cells.
- 4/ Perform the BsaI Golden Gate assembly to generate the integration vector of interest.
  We use a 20 fmol equimolar ratio of plasmids holding the DNA parts of interest. Here: pPOM041 (backbone), pPOM013 (promoter), pCOX (coding sequence), pYTK060 (tag) and pYTK064 (terminator).
  We then use 0.5 µL of the Golden Gate reaction mix for 20 µL of competent cells.
  pPOM041 uses AmpR as a selection marker, so *E. coli* cells can directly be plated on the selection media, without phenotype expression. pPOM041 also provides a GFP drop-out system, for green-white colony screening.
- 5/ To integrate the DNA sequence of interest, we digest 0.5–5 µg of the resulting plasmid with NotI and use it with the LiAc/PEG transformation procedure.

Example 2: Epitope tagging of a protein in the S. pombe *genome.*
This example replicates the experimental set-up of Moe et al, for the protein Cox4.[46] Our goal is to tag the C-ter of both Cox4 and Cox5 with TEV-6xGly-2xStrep.

Already proposed parts for use with POMBOX and MoClo-YTK
For use with POMBOX and MoClo-YTK, some parts are already available and ready to use:

- **pYTK089** is the backbone plasmid. It holds the *E. coli* origin of replication and an ampicillin resistance marker. It also bears a RFP drop-out system for red/white colony screening.
- **pYTK063**: with T*adh1* is selected as part type 4b. (terminator after Cter tag)
- **pYTK065:** with ConR1 as part type 5
- **pYTK077**: with KanR selection marker as part type 6.

New DNA parts have to be generated

The following parts are lacking in POMBOX and MoClo-YTK and have to be generated for this application:

- TEV-6xGly-2xStrep tag.
- 5′ Cox4
- 3′ Cox4

As in *example 1*, DNA sequences can be either synthesized as gene fragments or amplified from a template.
In both cases the key part of the design is related to the design of the overhangs.

TEV-6xGly-2xStrep tag is a regular part type 4a and therefore the overhangs can be as follows:

```
Flanking CGTCTCatcGGTCTCaATCC Part type 4a TGGCtGAGACCtGAGACG Flanking
          BsmBI     BsaI  Overhang     Overhang  BsaI   BsmBI
```

Cox4 3' is a regular part type 7, so the overhangs can be as follows:

```
Flanking CGTCTCatcGGTCTCaGAGT Part type 7  CCGAtGAGACCtGAGACG Flanking
          BsmBI     BsaI  Overhang     Overhang  BsaI   BsmBI
```

Cox4 5′ is a hybrid part, containing the 5′ overhang from part type 8b, and the 3′ overhang from part type 3. This way the only sequences that are inserted into the *S. pombe* genome are the TEV-6xGly-2xStrep tag, T*adh1*, ConnectorR1 and KanR.

```
Flanking CGTCTCatcGGTCTCaCAAT Part type 8a-3 ATCCtGAGACCtGAGACG Flanking
          BsmBI     BsaI  Overhang       Overhang  BsaI   BsmBI
```

- 3/ Store the DNA fragment in pYTK001. It uses BsmBI Golden Gate assembly, and pYTK001 possesses a GFP drop-out for green/white screen of transformant. If the DNA fragment was synthesized, we perform BsmBI Golden Gate assembly with a molar ratio of 1:1 of pYTK001 and the DNA fragment, 20 fmol. If the DNA fragment was obtained by amplification, we perform BsmBI Golden Gate assembly with 20 fmol of pYTK001 and 1 µL of the DNA fragment from gel clean up. We then use 0.5 µL of the Golden Gate reaction mix for 20 µL of competent cells.
- 4/ Perform the BsaI Golden Gate assembly to generate the integration vector of interest. We use a 20 fmol equimolar ratio of plasmids holding the DNA parts of interest. Here: pYTK089 (backbone), p5COX (5′ homology of Cox4), p3COX (3′ homology of Cox4), pYTK063 (terminator), pTEV-6xGly-2xStrep (tag), pYTK065 (connector), pYTK077 (selection marker). We then use 0.5 µL of the Golden Gate reaction mix for 20 µL of competent cells. pYTK089 uses AmpR as a selection marker, so *E. coli* cells can be directly plated on the selection media, without phenotype expression. pPOM041 also provides a GFP drop-out system for green-white colony screening.
- 5/ To integrate the DNA sequence of interest, we digest 0.5–5 µg of the resulting plasmid with NotI and use it with the LiAc/PEG transformation procedure.

List of overhangs:

BsmBI for pYTK001 ligation:	TCGG	sequence	GACC

BsaI for cassette plasmid assembly:

Part type 1, connector:	CCCT	sequence	AACG
Part type 2, promoter:	AACG	sequence	TATG
Part type 3, coding sequence:	TATG	sequence	ATCC
Part type 4, terminator:	ATCC	sequence	GCTG
Part type 5, connector:	GCTG	sequence	TACA
Part type 6, selection marker:	TACA	sequence	GAGT
Part type 7, 3' homology:	GAGT	sequence	CCGA
Part type 8a, Bacterial part:	CCGA	sequence	CAAT
Part type 8b, 5' homology:	CAAT	sequence	CCCT
Part type 3a, Nter tag:	TATG	sequence	TTCT
Part type 3b, coding sequence:	TTCT	sequence	ATCC
Part type 4a, Cter tag:	ATCC	sequence	TGGC
Part type 4b, terminator:	TGGC	sequence	GCTG

List of sequences

Overhang PCR primers were designed based on the Genome assembly ASM294v2 or plasmid sequences. The following overhang were added to primers:
Forward overhang 5′ actagacaacCGTCTCatcGGTCTCaNNNN 3′
Reverse overhang 5′ acaacacaacCGTCTCaGGTCTCaNNNN 3′

The PCR overhangs include Golden Gate overhangs related to the part type (NNNN), BsaI and BsmBI restriction sites.

Table S1: List of part plasmids generated for the POMBOX toolkit.

Plasmid name	Part type	Backbone & Selection marker	Description
pPOM001	1	pYTK001, Cm	Connector L6
pPOM002	1	pYTK001, Cm	Connector L7
pPOM003	1	pYTK001, Cm	Connector L8
pPOM004	1	pYTK001, Cm	Connector L9
pPOM005	1	pYTK001, Cm	Connector L10
pPOM006	1	pYTK001, Cm	Connector L11
pPOM007	5	pYTK001, Cm	Connector R6
pPOM008	5	pYTK001, Cm	Connector R7
pPOM009	5	pYTK001, Cm	Connector R8
pPOM010	5	pYTK001, Cm	Connector R9
pPOM011	5	pYTK001, Cm	Connector R10
pPOM012	5	pYTK001, Cm	Connector R11
pPOM013	2	pYTK001, Cm	Promoter *eno101*
pPOM014	2	pYTK001, Cm	Promoter *gpm1*
pPOM015	2	pYTK001, Cm	Promoter *adh1*
pPOM016	2	pYTK001, Cm	Promoter *rpl2501*
pPOM017	2	pYTK001, Cm	Promoter *tif*51
pPOM018	2	pYTK001, Cm	Promoter *rpl*701
pPOM019	2	pYTK001, Cm	Promoter *rpl*2102
pPOM020	2	pYTK001, Cm	Promoter *trs*1
pPOM021	2	pYTK001, Cm	Promoter *tub*1
pPOM022	2	pYTK001, Cm	Promoter *rps*1002
pPOM023	2	pYTK001, Cm	Promoter *apl*4
pPOM024	2	pYTK001, Cm	Promoter *mpa*1
pPOM025	2	pYTK001, Cm	Promoter *tup*11
pPOM026	2	pYTK001, Cm	Promoter *nmt*1
pPOM027	4	pUC57, Amp	Terminator *guo*
pPOM028	4	pUC57, Amp	Terminator *synth3*
pPOM029	4	pUC57, Amp	Terminator *synth25*
pPOM030	4	pUC57, Amp	Terminator *synth27*
pPOM031	4	pUC57, Amp	Terminator *synth29*
pPOM032	4	pUC57, Amp	Terminator *synth30*
pPOM033	7	pTwist, Amp	3'his5
pPOM034	7	pYTK001, Cm	3'lys3
pPOM035	7	pYTK001, Cm	3'ade6
pPOM036	7	pYTK001, Cm	3'ura4
pPOM037	8b	pYTK001, Cm	5'his5
pPOM038	8b	pTwist, Amp	5'lys3
pPOM039	8b	pYTK001, Cm	5'ade6
pPOM040	8b	pYTK001, Cm	5'ura4
pPOM041	15678	pYTK089, Amp	Integration vector for single-gene. *ura4* loci, KanR, LS/RE, GFP drop-out
pPOM042	15678	pYTK0091, Spec	Integration vector for multi-gene. *ura4* loci, KanR, LS'/RE', GFP drop-out

Table S2: List of P3 part (coding sequence) plasmids generated for this study.

Plasmid name	Part	Backbone & Selection marker	Description
pTP0013	P3	pYTK001, Cm	*At*PAL2
pTP0014	P3	pYTK001, Cm	*Aa*AMS1
pTP0017	P3	pYTK001, Cm	*Ca*MXMT1
pTP0209	P3	pYTK001, Cm	A0A0E3NXY0
pTP0210	P3	pYTK001, Cm	A0A5S9IQ85
pTP0211	P3	pYTK001, Cm	A0A653G212
pTP0214	P3	pYTK001, Cm	A0A8I0Q8N9
pTP0215	P3	pYTK001, Cm	C4ZEM0
pTP0216	P3	pYTK001, Cm	UPI41561
pTP0217	P3	pYTK001, Cm	A0A8J2KXK4
pTP0219	P3	pYTK001, Cm	A0A1G0GBF6
pTP0220	P3	pYTK001, Cm	A0A8J2P3A2
pTP0221	P3	pYTK001, Cm	F0ZD71
pTP0222	P3	pYTK001, Cm	A0A1I7GSV9
pTP0223	P3	pYTK001, Cm	A0A348B793

Table S3: List of MoClo-YTK part plasmids used in this study.

Plasmid name	Part type	Description
pYTK001	Backbone	Part Plasmid Entry Vector
pYTK002	1	ConLS
pYTK003	1	ConL1
pYTK004	1	ConL2
pYTK005	1	ConL3
pYTK006	1	ConL4
pYTK007	1	ConL5
pYTK008	1	ConLS'
pTYK009	2	P*tdh3*
pYTK010	2	P*ccw12*
pYTK011	2	P*pgk1*
pYTK012	2	P*hhf2*
pYTK013	2	P*tef1*
pYTK014	2	P*tef2*
pTYK015	2	P*hhf1*
pYTK016	2	P*htb2*
pYTK017	2	P*rpl18b*
pYTK018	2	P*ald6*
pYTK019	2	P*pab1*
pYTK020	2	P*ret2*
pYTK033	3	Venus
pYTK034	3	mRuby2
pYTK047	234	GFP dropout
pYTK051	4	T*eno1*
pYTK052	4	T*ssa1*
pYTK053	4	T*adh1*
pYTK054	4	T*pgk1*
pYTK055	4	T*eno2*
pYTK056	4	T*tdh1*
pYTK067	5	ConR1
pYTK068	5	ConR2
pYTK069	5	ConR3
pYTK070	5	ConR4
pYTK071	5	ConR5
pYTK072	5	ConRE
pYTK073	5	ConRE'
pYTK077	6	KanamycinR
pYTK089	8a	AmpR-ColE1
pYTK091	8a	SpecR-ColE1

Table S4: List of single-gene expression plasmids generated for this study.

Plasmid name	Parts used	Backbone & Selection marker	Description
pTP0024	pPOM015, pYTK0034, pPOM027	pPOM041, Amp	P*adh1*, mRuby2, T*guo*
pTP0025	pPOM015, pYTK0034, pPOM028	pPOM041, Amp	P*adh1,* mRuby2, T*synth3*
pTP0026	pPOM015, pYTK0034, pPOM029	pPOM041, Amp	P*adh1,* mRuby2, T*synth25*
pTP0028	pPOM015, pYTK0034, pPOM030	pPOM041, Amp	P*adh1,* mRuby2, T*synth27*
pTP0029	pPOM015, pYTK0034, pPOM031	pPOM041, Amp	P*adh1,* mRuby2, T*synth29*
pTP0030	pPOM015, pYTK0034, pPOM032	pPOM041, Amp	P*adh1,* mRuby2, T*synth30*
pTP0090	pPOM015, pYTK0034, pYTK052	pPOM041, Amp	Padh1, mRuby2, T*ssa1*
pTP0091	pPOM015, pYTK0034, pYTK053	pPOM041, Amp	P*adh1*, mRuby2, T*adh1*
pTP0092	pPOM015, pYTK0034, pYTK054	pPOM041, Amp	P*adh1*, mRuby2, T*pgk1*
pTP0093	pPOM015, pYTK0034, pYTK055	pPOM041, Amp	P*adh1*, mRuby2, T*eno2*
pTP0094	pPOM015, pYTK0034, pYTK056	pPOM041, Amp	P*adh1*, mRuby2, T*tdh1*
pTP0133	pPOM026, pYTK0034, pYTK051	pPOM041, Amp	P*nmt1*, mRuby2, T*eno1*
pTP0134	pPOM015, pYTK0034, pYTK051	pPOM041, Amp	P*adh1*, mRuby2, T*eno1*
pTP0135	pPOM021, pYTK0034, pYTK051	pPOM041, Amp	P*tub1*, mRuby2, T*eno1*
pTP0136	pPOM017, pYTK0034, pYTK051	pPOM041, Amp	P*tif51*, mRuby2, T*eno1*
pTP0138	pPOM014, pYTK0034, pYTK051	pPOM041, Amp	P*gpm1*, mRuby2, T*eno1*
pTP0139	pPOM013, pYTK0034, pYTK051	pPOM041, Amp	P*eno101*, mRuby2, T*eno1*
pTP0140	pPOM022, pYTK0034, pYTK051	pPOM041, Amp	P*rps1002*, mRuby2, Teno1
pTP0142	pPOM019, pYTK0034, pYTK051	pPOM041, Amp	P*rpl2102*, mRuby2, Teno1
pTP0143	pPOM018, pYTK0034, pYTK051	pPOM041, Amp	P*rpl701*, mRuby2, T*eno1*
pTP0145	pPOM016, pYTK0034, pYTK051	pPOM041, Amp	P*rpl2501*, mRuby2, T*eno1*
pTP0146	pPOM020, pYTK0034, pYTK051	pPOM041, Amp	P*trs1*, mRuby2, T*eno1*
pTP0148	pPOM023, pYTK0034, pYTK051	pPOM041, Amp	P*apl4*, mRuby2, T*eno1*
pTP0149	pPOM025, pYTK0034, pYTK051	pPOM041, Amp	P*tup11*, mRuby2, T*eno1*
pTP0151	pPOM024, pYTK0034, pYTK051	pPOM041, Amp	P*mpa1*, mRuby2, T*eno1*
pTP0159	pPOM026, pYTK0033, pYTK051	pPOM041, Amp	P*nmt1*, Venus, T*eno1*
pTP0160	pPOM015, pYTK0033, pYTK051	pPOM041, Amp	P*adh1*, Venus, T*eno1*
pTP0161	pPOM021, pYTK0033, pYTK051	pPOM041, Amp	P*tub1*, Venus, T*eno1*
pTP0162	pPOM017, pYTK0033, pYTK051	pPOM041, Amp	P*tif51*, Venus, T*eno1*
pTP0164	pPOM014, pYTK0033, pYTK051	pPOM041, Amp	P*gpm1*, Venus, T*eno1*
pTP0165	pPOM013, pYTK0033, pYTK051	pPOM041, Amp	P*eno101*, Venus, T*eno1*
pTP0166	pPOM022, pYTK0033, pYTK051	pPOM041, Amp	P*rps1002*, Venus, T*eno1*
pTP0168	pPOM019, pYTK0033, pYTK051	pPOM041, Amp	P*rpl2102*, Venus, T*eno1*
pTP0169	pPOM018, pYTK0033, pYTK051	pPOM041, Amp	P*rpl701*, Venus, T*eno1*
pTP0171	pPOM016, pYTK0033, pYTK051	pPOM041, Amp	P*rpl2501*, Venus, T*eno1*
pTP0172	pPOM020, pYTK0033, pYTK051	pPOM041, Amp	P*trs1*, Venus, T*eno1*
pTP0174	pPOM023, pYTK0033, pYTK051	pPOM041, Amp	P*apl4*, Venus, T*eno1*
pTP0175	pPOM025, pYTK0033, pYTK051	pPOM041, Amp	P*tup11*, Venus, T*eno1*
pTP0177	pPOM024, pYTK0033, pYTK051	pPOM041, Amp	P*mpa1*, Venus, T*eno1*
pTP0206	pPOM015, pTP0017, pYTK051	pPOM041, Amp	P*adh1*, AtPAL2, T*eno1*
pTP0207	pPOM015, pTP0014, pYTK051	pPOM041, Amp	P*adh1*, CaMXMT1*, Teno1*
pTP0208	pPOM015, pYTK0033, pYTK051	pPOM041, Amp	P*adh1*, AaAMS, T*eno1*
pTP0265	pYTK002, pYTK009, pTP0209, pYTK0051, pYTK067	pYTK095, Amp	ConLS, P*tdh3*, A0A0E3NXY0, *Teno1*, ConR1
pTP0266	pYTK003, pYTK010, pTP0210, pYTK0052, pYTK072	pYTK095, Amp	ConL1, P*ccw12*, A0A5S9IQ85, T*ssa1*, ConRE
pTP0267	pYTK003, pYTK010, pTP0210, pYTK0052, pYTK06	pYTK095, Amp	ConL1, P*ccw12*, A0A5S9IQ85, T*ssa1*, ConR2
pTP0269	pYTK004, pYTK011, pTP0211, pYTK0053, pYTK069	pYTK095, Amp	ConL2, P*pgk1*, A0A653G212, T*adh1*, ConR3

pTP0270	pYTK005, pYTK012, pTP0214, pYTK0054, pYTK070	pYTK095, Amp	ConL3, P*hhf2*, A0A8I0Q8N9, T*pgk1*, ConRE
pTP0271	pYTK005, pYTK012, pTP0214, pYTK0054, pYTK070	pYTK095, Amp	ConL3, P*hhf2*, A0A8I0Q8N9, T*pgk1*, ConR4
pTP0273	pYTK006, pYTK013, pTP0215, pYTK0055, pYTK071	pYTK095, Amp	ConL4, P*tef1*, C4ZEM0, T*eno2*, ConR5
pTP0274	pYTK007, pYTK014, pTP0216, pYTK0056, pYTK072	pYTK095, Amp	ConL5, P*tef2*, UPI41561, T*tdh1*, ConRE
pTP0275	pYTK007, pYTK014, pTP0216, pYTK0056 // pPOM007	pYTK095, Amp	ConL5, P*tef2*, UPI41561, T*tdh1*, ConR6
pTP0277	pPOM001, pYTK015, pTP0217, pPOM027, pPOM008	pYTK095, Amp	ConL6, P*hhf1*, A0A8J2KXK4, T*guo*, ConR7
pTP0278	pPOM002, pYTK016, pTP0223, pPOM028, pYTK072	pYTK095, Amp	ConL7, P*htb2*, A0A1G0GBF6, T*synth3*, ConRE
pTP0279	pPOM002, pYTK016, pTP0223, pPOM028, pPOM009	pYTK095, Amp	ConL7, P*htb2*, A0A1G0GBF6, T*synth3*, ConR8
pTP0281	pPOM003, pYTK017, pTP0219, pPOM029, pPOM010	pYTK095, Amp	ConL8, P*rpl18b*, A0A8J2P3A2, T*synth25*, ConR9
pTP0282	pPOM004, ppYTK018, pTP0220, pPOM030, ppYTK072	pYTK095, Amp	ConL9, P*ald6*, F0ZD71, T*synth27*, ConRE
pTP0283	pPOM004, pYTK018, pTP0220, pPOM030, pPOM011	pYTK095, Amp	ConL9, P*ald6*, F0ZD71, T*synth27*, ConR10
pTP0285	pPOM005, pYTK019, pTP0221, pPOM031, pPOM012	pYTK095, Amp	ConL10, P*pab1*, A0A1I7GSV9, T*synth29*, ConR11
pTP0286	pPOM006, pYTK020, pTP0222, pPOM032, pYTK072	pYTK095, Amp	ConL11, P*ret2*, A0A348B793, T*synth30*, ConRE

Table S5: Multi-gene plasmids generated for this study.

Plasmid name	Parts used	Backbone & Selection marker	Description
pTP0287	pTP0265, pTP0266	pPOM042, Spec	multi-gene plasmids, 2 transcriptional units
pTP0288	pTP0265, pTP0267, pTP0269, pTP0270	pPOM042, Spec	multi-gene plasmids, 4 transcriptional units
pTP0289	pTP0265, pTP0267, pTP0269, pTP0271, pTP0273, pTP0274	pPOM042, Spec	multi-gene plasmids, 6 transcriptional units
pTP0290	pTP0265, pTP0267, pTP0269, pTP0271, pTP0273, pTP0275, pTP0277, pTP0278	pPOM042, Spec	multi-gene plasmids, 8 transcriptional units
pTP0291	pTP0265, pTP0267, pTP0269, pTP0271, pTP0273, pTP0275, pTP0277, pTP0279, pTP0281, pTP0282	pPOM042, Spec	multi-gene plasmids, 10 transcriptional units
pTP0292	pTP0265, pTP0267, pTP0269, pTP0271, pTP0273, pTP0275, pTP0277, pTP0279, pTP0281, pTP0283, pTP0285, pTP0286	pPOM042, Spec	multi-gene plasmids, 12 transcriptional units

Table S6: List of *S. pombe* strains used in this study.

Name	Genotype	Ref	Used in
h- 972		Leupold U[47]	Figure 3, 4, 5, S2
h+ *975 ura4*	ura4-D18	Public domain	Figure 6
Spo_pTP0024	h+ *975* ura4-D18 [ura4+] P*adh1*::mRuby2::T*guo* [G418R]	This study	Figure 4
Spo_pTP0025	h+ 975 ura4-D18 [ura4+] Padh1::mRuby2::T*synth3*[G418R]	This study	Figure 4
Spo_pTP0026	h+ 975 ura4-D18 [ura4+] adh1::mRuby2::T*synth25*[G418R]	This study	Figure 4
Spo_pTP0028	h+ 975 ura4-D18 [ura4+] P*adh1*::mRuby2::T*synth27* [G418R]	This study	Figure 4
Spo_pTP0029	h+ 975 ura4-D18 [ura4+] P*adh1*::mRuby2::T*synth29* [G418R]	This study	Figure 4
Spo_pTP0030	h+ 975 ura4-D18 [ura4+] P*adh1*::mRuby2::T*synth30* [G418R]	This study	Figure 4
Spo_pTP0090	h+ 975 ura4-D18 [ura4+] P*adh1*::mRuby2::T*ssa1*[G418R]	This study	Figure 4
Spo_pTP0091	h+ 975 ura4-D18 [ura4+] P*adh1*::mRuby2::T*adh1*[G418R]	This study	Figure 4
Spo_pTP0092	h+ 975 ura4-D18 [ura4+] P*adh1*::mRuby2::T*pgk1* [G418R]	This study	Figure 4
Spo_pTP0093	h+ 975 ura4-D18 [ura4+] P*adh1*::mRuby2::T*eno2* [G418R]	This study	Figure 4
Spo_pTP0094	h+ 975 ura4-D18 [ura4+] P*adh1*::mRuby2::T*tdh1* [G418R]	This study	Figure 4
Spo_pTP0133	h+ 975 ura4-D18 [ura4+] P*nmt1*::mRuby2::T*eno1* [G418R]	This study	Figure 3
Spo_pTP0134	h+ 975 ura4-D18 [ura4+] P*adh1*::mRuby2::T*eno1* [G418R]	This study	Figure 3, 4
Spo_pTP0135	h+ 975 ura4-D18 [ura4+] P*tub1*::mRuby2::T*eno1* [G418R]	This study	Figure 3
Spo_pTP0136	h+ 975 ura4-D18 [ura4+] P*tif51*::mRuby2::T*eno1* [G418R]	This study	Figure 3
Spo_pTP0138	h+ 975 ura4-D18 [ura4+] P*gpm1*::mRuby2::T*eno1* [G418R]	This study	Figure 3
Spo_pTP0139	h+ 975 ura4-D18 [ura4+] P*eno101*::mRuby2::T*eno1* [G418R]	This study	Figure 3
Spo_pTP0140	h+ 975 ura4-D18 [ura4+] P*rps1002*::mRuby2::T*eno1* [G418R]	This study	Figure 3
Spo_pTP0142	h+ 975 ura4-D18 [ura4+] P*rpl2102*::mRuby2::T*eno1* [G418R]	This study	Figure 3
Spo_pTP0143	h+ 975 ura4-D18 [ura4+] P*rpl701*::mRuby2::T*eno1* [G418R]	This study	Figure 3
Spo_pTP0145	h+ 975 ura4-D18 [ura4+] P*rpl2501*::mRuby2::T*eno1* [G418R]	This study	Figure 3
Spo_pTP0146	h+ 975 ura4-D18 [ura4+] P*trs1*::mRuby2::T*eno1* [G418R]	This study	Figure 3
Spo_pTP0148	h+ 975 ura4-D18 [ura4+] P*apl4*::mRuby2::T*eno1* [G418R]	This study	Figure 3
Spo_pTP0149	h+ 975 ura4-D18 [ura4+] P*tup11*::mRuby2::T*eno1* [G418R]	This study	Figure 3
Spo_pTP0151	h+ 975 ura4-D18 [ura4+] P*mpa1*::mRuby2::T*eno1* [G418R]	This study	Figure 3
Spo_pTP0159	h+ 975 ura4-D18 [ura4+] P*nmt1*::Venus::T*eno1* [G418R]	This study	Figure 3
Spo_pTP0160	h+ 975 ura4-D18 [ura4+] P*adh1*::Venus::T*eno1* [G418R]	This study	Figure 3
Spo_pTP0161	h+ 975 ura4-D18 [ura4+] P*tub1*::Venus::T*eno1* [G418R]	This study	Figure 3
Spo_pTP0162	h+ 975 ura4-D18 [ura4+] P*tif51*::Venus::T*eno1* [G418R]	This study	Figure 3
Spo_pTP0164	h+ 975 ura4-D18 [ura4+] P*gpm1*::Venus::T*eno1* [G418R]	This study	Figure 3
Spo_pTP0165	h+ 975 ura4-D18 [ura4+] P*eno101*::Venus::T*eno1* [G418R]	This study	Figure 3
Spo_pTP0166	h+ 975 ura4-D18 [ura4+] P*rps1002*::Venus::T*eno1* [G418R]	This study	Figure 3
Spo_pTP0168	h+ 975 ura4-D18 [ura4+] P*rpl2102*::Venus::T*eno1* [G418R]	This study	Figure 3
Spo_pTP0169	h+ 975 ura4-D18 [ura4+] P*rpl701*::Venus::T*eno1* [G418R]	This study	Figure 3
Spo_pTP0171	h+ 975 ura4-D18 [ura4+] P*rpl2501*::Venus::T*eno1* [G418R]	This study	Figure 3
Spo_pTP0172	h+ 975 ura4-D18 [ura4+] P*trs1*::Venus::T*eno1* [G418R]	This study	Figure 3
Spo_pTP0174	h+ 975 ura4-D18 [ura4+] P*apl4*::Venus::T*eno1* [G418R]	This study	Figure 3
Spo_pTP0175	h+ 975 ura4-D18 [ura4+] P*tup11*::Venus::T*eno1* [G418R]	This study	Figure 3
Spo_pTP0177	h+ 975 ura4-D18 [ura4+] P*mpa1*::Venus::T*eno1* [G418R]	This study	Figure 3
Spo_pTP0206	h+ 975 ura4-D18 [ura4+] P*adh1*::AtPAL2::T*eno1* [G418R]	This study	Figure 5
Spo_pTP0207	h+ 975 ura4-D18 [ura4+] P*adh1*::CaMXMT1::T*eno1* [G418R]	This study	Figure 5
Spo_pTP0208	h+ 975 ura4-D18 [ura4+] P*adh1*::AaAMS::T*eno1* [G418R]	This study	Figure 5
Spo_pTP0287	h+ 975 ura4-D18 [ura4+] pTP0265::pTP0266 [G418R]	This study	Figure 6
Spo_pTP0288	h+ 975 ura4-D18 [ura4+] pTP0265::pTP0267::pTP0269::pTP0270 [G418R]	This study	Figure 6
Spo_pTP0289	h+ 975 ura4-D18 [ura4+] pTP0265::pTP0267::pTP0269::pTP0271::pTP0273::pTP0274 [G418R]	This study	Figure 6
Spo_pTP0290	h+ 975 ura4-D18 [ura4+] pTP0265::pTP0267::pTP0269::pTP0271::pTP0273::pTP0275::p	This study	Figure 6

	TP0277::pTP0278[G418R]		
Spo_pTP0291	h+ 975 ura4-D18 [ura4+] pTP0265::pTP0267::pTP0269::pTP0271::pTP0273::pTP0275::pTP0277::pTP0279::pTP0281::pTP0282 [G418R]	This study	Figure 6
Spo_pTP0292	h+ 975 ura4-D18 [ura4+] pTP0265::pTP0267::pTP0269::pTP0271::pTP0273::pTP0275::pTP0277::pT H016::pTP0281::pTP0283::pTP0285::pTP0286 [G418R]	This study	Figure 6

Table S7: List of genotyping and sequencing primers.

Name	Sequence	Target	Use
**TH0033F**	ttgctggccttttgctcacatg	ColE1	Sanger sequencing, Colony PCR
**TH0033R**	acctcagaactccatctggatttgttcag	CamR terminator	Sanger sequencing, Colony PCR
**TH0041F**	aagactgtcaaggagggtattctggg	AgTEF promoter	Sanger sequencing, Colony PCR
**TH0042F**	gcctcgaaacgtgagtcttttcc	KanR	Sanger sequencing, Colony PCR
**TH0116R**	gataggtgcctcactgattaagcattgg	AmpR terminator	Sanger sequencing, Colony PCR
**TH0104F**	gtgtgtactttgaaagtctagctttacagcttgg	Ura4_3''	Chromosomal integration verification
**TH0104R**	gtcaatcgtatgtgaatgctggtcgc	Ura4_3''	Chromosomal integration verification
**TH0117F**	gcttgtaccggctgtttgtgg	Ura4_5'	Chromosomal integration verification
**TH0117R**	cctctaaaaaagttgatgccagaccg	Ura4_5'	Chromosomal integration verification
**TH0119**	agcagttacagagatgttacgaacc	ConE scar 5'	Chromosomal integration verification
**TH0120**	ggttcgtaacatctctgtaactgct	ConE scar 3'	Chromosomal integration verification
**TH0121**	ctgaactggccgataattgcagacg	ConS scar 5'	Chromosomal integration verification
**TH0122**	cgtctgcaattatcggccagttcag	ConS scar 3'	Chromosomal integration verification
**TH0123**	ccaaaccagatgtcaacacagctac	Con1 scar 5'	Chromosomal integration verification
**TH0124**	gtagctgtgttgacatctggtttgg	Con1 scar 3'	Chromosomal integration verification
**TH0125**	cacacactggcttaagatgac	Con2 scar 5'	Chromosomal integration verification
**TH0126**	gtcatcttaagccagtgtgtg	Con2 scar 3'	Chromosomal integration verification
**TH0127**	gttctgtatgggcacagacaaccta	Con3 scar 5'	Chromosomal integration verification
**TH0128**	taggttgtctgtgcccatacagaac	Con3 scar 3'	Chromosomal integration verification
**TH0129**	ggtagactacccatgagtcacaatg	Con4 scar 5'	Chromosomal integration verification
**TH0130**	cattgtgactcatgggtagtctacc	Con4 scar 3'	Chromosomal integration verification
**TH0131**	aagtgaataaagctccacacagtcg	Con5 scar 5'	Chromosomal integration verification
**TH0132**	cgactgtgtggagctttattcactt	Con5 scar 3'	Chromosomal integration verification
**TH0133**	gctgagtcatcacgctaca	Con6 scar 5'	Chromosomal integration verification
**TH0134**	tgtagcgtgatgactcagc	Con6 scar 3'	Chromosomal integration verification
**TH0135**	gctgcgagctagagcgtac	Con7 scar 5'	Chromosomal integration verification
**TH0136**	gtacgctctagctcgcagc	Con7 scar 3'	Chromosomal integration verification
**TH0137**	gctgaccgggtactgaaag	Con8 scar 5'	Chromosomal integration verification
**TH0138**	ctttcagtacccggtcagc	Con8 scar 3'	Chromosomal integration verification
**TH0139**	gctgttttgtcggcctaca	Con9 scar 5'	Chromosomal integration verification
**TH0140**	tgtaggccgacaaaacagc	Con9 scar 3'	Chromosomal integration verification
**TH0141**	gctgattgacgttccatat	Con10 scar 5'	Chromosomal integration verification
**TH0142**	atatggaacgtcaatcagc	Con10 scar 3'	Chromosomal integration verification
**TH0143**	gctgttaccacagccaacg	Con11 scar 5'	Chromosomal integration verification
**TH0144**	cgttggctgtggtaacagc	Con11 scar 3'	Chromosomal integration verification
**TH0171**	actgcctggcgactcacaac	ConRE	Chromosomal integration verification
**TH0172**	cgctcggatgactgcatgatgac	ConR1	Chromosomal integration verification
**TH0173**	catcgtatctcacaggagcaagcg	ConR2	Chromosomal integration verification
**TH0174**	gcacgctcatatcatcgtcgtc	ConR3	Chromosomal integration verification
**TH0175**	ggtgacaacgctggacagatgac	ConR4	Chromosomal integration verification
**TH0176**	aatagtgccgatgaccacagacc	ConR5	Chromosomal integration verification
**TH0177**	ggcgttgctcattaggtaccgg	ConR6	Chromosomal integration verification
**TH0178**	ctcgtggctttatcgagcggatc	ConR7	Chromosomal integration verification
**TH0179**	ccgagcgatgtgaaggactcag	ConR8	Chromosomal integration verification
**TH0180**	ggacttccagttctgcagcgac	ConR9	Chromosomal integration verification
**TH0181**	ccctatccctatataccgcaactttgtg	ConR10	Chromosomal integration verification
**TH0182**	gttgtcaatggctttcaggcttgc	ConR11	Chromosomal integration verification

Table S8: List of primers for DNA part construction.

Name	Sequence	Target	
**TH0001F**	actagactagacaaccgtctcatcggtctcaaacgtcgccataaaagacagaataagtc	P*nmt1*, *S. pombe* genome,	(1), (3)
**TH0001R**	ctatttacgtgtcatacgcaaagtc	P*nmt1*, *S. pombe* genome	(2), (3)
**TH0002F**	gactttgcgtatgacacgtaaatag	P*nmt1*, *S. pombe* genome	(2), (3)
**TH0002R**	gttgtgttgtgttgtcgtctcaggtctcacatagatcttttaacaaagcgactataagtcag	P*nmt1*, *S. pombe* genome	(1), (3)
**TH0011F**	actagactagacaaccgtctcatcggtctcaaacgatcctcattctttggagccc	P*tub1*, pDUAL_FFH21	(1)
**TH0011R**	acaacacaacacaaccgtctcaggtctcacatagatctgaacgtctttccagggtttc	P*tub1*, pDUAL_FFH21	(1)
**TH0013F**	actagactagacaaccgtctcatcggtctcaaacgaaccggactcatctttgtg	P*tif51*, pDUAL_FFH51	(1)
**TH0013R**	acaacacaacacaaccgtctcaggtctcacatagatcttctaaacgattcttaaacttgaagag	P*tif51*, pDUAL_FFH51	(1)
**TH0039F**	actagactagacaaccgtctcatcggtctcaaacggccctacaacaactaagaaaatggctatc	P*adh1*, *S. pombe* genome	(1), (3)
**TH0039R**	gaccaatgagtcgcggaaggggagc	P*adh1*, *S. pombe* genome	(2), (3)
**TH0040F**	tccccttccgcgactcattggtc	P*adh1*, *S. pombe* genome	(2), (3)
**TH0040R**	acaacacaacacaaccgtctcaggtctcacatagatctaattctcttgcttaaagaaaagcgaaggcac	P*adh1*, *S. pombe* genome	(1), (3)
**TH0043F**	actagactagacaaccgtctcatcggtctcagagtttattattcaaaactagtatactttttctcgg	ura4_3'', *S. pombe* genome	(1)
**TH0043R**	acaacacaacacaaccgtctcaggtctcatcggtgctctgtacacgtattctttcc	ura4_3'', *S. pombe* genome	(1)
**TH0044F**	actagactagacaaccgtctcatcggtctcacaatatgcttacataatgagtgagtg	ura4_5', *S. pombe* genome	(1), (3)
**TH0044R**	caaaagcaagagtccacgtcc	ura4_5', *S. pombe* genome	(2), (3)
**TH0045F**	ggacgtggactcttgcttttg	ura4_5', *S. pombe* genome	(2), (3)
**TH0045R**	gcccgtcacctttaacatcca	ura4_5', *S. pombe* genome	(2), (3)
**TH0046F**	tggatgttaaaggtgacgggc	ura4_5', *S. pombe* genome	(2), (3)
**TH0046R**	acaacacaacacaaccgtctcaggtctcaagggaagcttgtgatattgacgaaac	ura4_5', *S. pombe* genome	(1), (3)
**TH0047F**	actagacaaccgtctcatcggtctcagagtagaatcctgaataatgtgctgtg	ade6_3'', *S. pombe* genome	(1)
**TH0047R**	acaacacaacacaaccgtctcaggtctcatcggaaacataatgcggtccgatatg	ade6_3'', *S. pombe* genome	(1)
**TH0048F**	actagactagacaaccgtctcatcggtctcacaataaacgttgctttatatatggataacg	ade6_5', *S. pombe* genome	(1), (3)
**TH0048R**	tgaatggtttcagttgtaggataagc	ade6_5', *S. pombe* genome	(2), (3)
**TH0049F**	gcttatcctacaactgaaaccattca	ade6_5', *S. pombe* genome	(2), (3)
**TH0049R**	gagtaaaatggtttcaccatcttgc	ade6_5', *S. pombe* genome	(2), (3)
**TH0050F**	gcaagatggtgaaaccattttactc	ade6_5', *S. pombe* genome	(2), (3)
**TH0050R**	acaacacaaccgtctcaggtctcaagggttttaattgcgtcgcagc	ade6_5', *S. pombe* genome	(1), (3)
**TH0051F**	actagactagacaaccgtctcatcggtctccagagccaaatacgatgaccagtcctgccttacgaagttaaattatagg	lys3_3'', *S. pombe* genome	(1), (3)
**TH0051R**	cgttaaaaggtgtcaaatatggtttcatg	lys3_3'', *S. pombe* genome	(2), (3)
**TH0052F**	catgaaaccatatttgacaccttttaacg	lys3_3'', *S. pombe* genome	(2), (3)
**TH0052R**	acaacacaacacaaccgtctcaggtctcatcggtactgtccttaatcttcagttataatgac	lys3_3'', *S. pombe* genome	(1), (3)
**TH0053F**	actagactagacaaccgtctcatcggtctcacaatgggatgccaaagttagatcctactagtaaaaatcaagtgaattgtgc	lys3_5', *S. pombe* genome	(1), (3)

**TH0053R**	gtggcgaaggagagcaaaaacttaatc	lys3_5', *S. pombe* genome	(2), (3)
**TH0054F**	gattaagtttttgctctccttcgccac	lys3_5', *S. pombe* genome	(2), (3)
**TH0054R**	ggaagctctttgagtccaataatgtac	lys3_5', *S. pombe* genome	(2), (3)
**TH0055F**	gtacattattggactcaaagagcttcc	lys3_5', *S. pombe* genome	(2), (3)
**TH0055R**	acaacacaacacaaccgtctcaggtctcaagggaagcaataaatattggggaaag	lys3_5', *S. pombe* genome	(1), (3)
**TH0056F**	actagactagacaaccgtctcatcggtctcacaatgttaccatgggagtacgttcgtcctcaattcgatgttgcagattttac	his5_5', *S. pombe* genome	(1)
**TH0056R**	acaacacaacacaaccgtctcaggtctcaagggtggtataaacaataaatacaccaaggataaaaaataagaaatcag	his5_5', *S. pombe* genome	(1)
**TH0057F**	acaacacaacacaaccgtctcaggtctcatcggtcctccactccaaagcgaaaattaattc	his5_3'', *S. pombe* genome	(1), (3)
**TH0057R**	ctggcttaaagcaatgaggttgtaattaaatggcttgagagctagaac	his5_3'', *S. pombe* genome	(2), (3)
**TH0058F**	caacctcattgctttaagccagactgcaattggtaaaagagagcattgg	his5_3'', *S. pombe* genome	(2), (3)
**TH0058R**	actagactagacaaccgtctcatcggtctcagagtatggaacgattgaatactgtgctactctttatg	his5_3'', *S. pombe* genome	(1), (3)
**TH0084F**	actagacaaccgtctcatcggtctcaaacgcttttccacctgcaaacgc	SPAC26F1.06, P*gpm1*, *S. pombe* genome	(1)
**TH0153**	gtgcatatgcgtctcaggtctcacatagatctcgttaacgttgtatagtgtggtgtaag	SPAC26F1.06, P*gpm1*, *S. pombe* genome	(1)
**TH0085F**	actagacaaccgtctcatcggtctcaaacggattctcttgcaactgcctgc	SPBC1815.01, P*eno101*, *S. pombe* genome	(1)
**TH0154**	gtgcatatgcgtctcaggtctcacatagatctgattaacgatgtttactgtagaatactaattg	SPBC1815.01, P*eno101*, *S. pombe* genome	(1)
**TH0088F**	actagacaaccgtctcatcggtctcaaacgcgttgacaagactggcgag	SPAC959.08, P*rpl2102*, *S. pombe* genome	(1)
**TH0157**	gtgcatatgcgtctcaggtctcacatagatcttgctgttcctggttaaaggc	SPAC959.08, P*rpl2102*, *S. pombe* genome	(1)
**TH0089F**	actagacaaccgtctcatcggtctcaaacgcaaagctctccttatacaccgatg	SPBC18H10.12c, P*rpl701*, *S. pombe* genome	(1)
**TH0158**	gtgcatatgcgtctcaggtctcacatagatctgatggtggatgtctggtgg	SPBC18H10.12c, P*rpl701*, *S. pombe* genome	(1)
**TH0091F**	actagacaaccgtctcatcggtctcaaacgctcctgtgcttgatctacgcaag	SPBC106.18, P*rpl2501*, *S. pombe* genome	(1)
**TH0160**	gtgcatatgcgtctcaggtctcacatagatctgatgtcctgtcaattaagagttggttgttgctcg	SPBC106.18, P*rpl2501*, *S. pombe* genome	(1)
**TH0094F**	actagacaaccgtctcatcggtctcaaacgccaaatgactgacaggtac	SPCP1E11.06, P*apl4*, *S. pombe* genome	(1)
**TH0162**	gtgcatatgcgtctcaggtctcacatagatctctttggatgcgtctttgg	SPCP1E11.06, P*apl4*, *S. pombe* genome	(1)
**TH0095F**	actagacaaccgtctcatcggtctcaaacgtaacaagatatgcaatgaacgttgtccgtc	SPAC18B11.10, P*tup11*, *S. pombe* gneome	(1)
**TH0163**	gtgcatatgcgtctcaggtctcacatagatcttttttttttcagtcagacttattgc	SPAC18B11.10, P*tup11*, *S. pombe* genome	(1)
**TH0097F**	actagacaaccgtctcatcggtctcaaacggagcaaattcagcagcttcctcac	SPAPB1E7.11c, P*mpa1*, *S. pombe* genome	(1)
**TH0097R**	acaacacaaccgtctcaggtctcacatagatctgtatactaggggtttctgttagcgatttac	SPAPB1E7.11c, P*mpa1*, *S. pombe* genome	(1)

(1): BsmBI and BsaI overhang, (2): single base mutation to discard BsmBI site, (3) overlap PCR

Supplementary References

(46) MoeA.ÄdelrothP.BrzezinskiP.Näsvik ÖjemyrL.Cryo-EM Structure and Function of S. Pombe Complex IV with Bound Respiratory Supercomplex FactorCommun. Chem.2023613210.1038/s42004-023-00827-3
(47) Leupold, U. Die Vererbung von Homothallie und Heterothallie bei Schizosaccharomyces Pombe; Luno, 1950; Vol. 24, pp 381–480.
